# In silico study of multicellular automaticity of heterogeneous cardiac cell monolayers: Effects of automaticity strength and structural linear anisotropy

**DOI:** 10.1371/journal.pcbi.1005978

**Published:** 2018-03-12

**Authors:** James Elber Duverger, Vincent Jacquemet, Alain Vinet, Philippe Comtois

**Affiliations:** 1 Research Centre, Montreal Heart Institute, Montreal, Quebec, Canada; 2 Department of Pharmacology and Physiology / Institute of Biomedical Engineering, Université de Montréal, Montreal, Quebec, Canada; 3 Research Centre, Hôpital du Sacré-Coeur de Montréal, Montreal, Quebec, Canada; University of California San Diego, UNITED STATES

## Abstract

The biological pacemaker approach is an alternative to cardiac electronic pacemakers. Its main objective is to create pacemaking activity from added or modified distribution of spontaneous cells in the myocardium. This paper aims to assess how automaticity strength of pacemaker cells (i.e. their ability to maintain robust spontaneous activity with fast rate and to drive neighboring quiescent cells) and structural linear anisotropy, combined with density and spatial distribution of pacemaker cells, may affect the macroscopic behavior of the biological pacemaker. A stochastic algorithm was used to randomly distribute pacemaker cells, with various densities and spatial distributions, in a semi-continuous mathematical model. Simulations of the model showed that stronger automaticity allows onset of spontaneous activity for lower densities and more homogeneous spatial distributions, displayed more central foci, less variability in cycle lengths and synchronization of electrical activation for similar spatial patterns, but more variability in those same variables for dissimilar spatial patterns. Compared to their isotropic counterparts, *in silico* anisotropic monolayers had less central foci and displayed more variability in cycle lengths and synchronization of electrical activation for both similar and dissimilar spatial patterns. The present study established a link between microscopic structure and macroscopic behavior of the biological pacemaker, and may provide crucial information for optimized biological pacemaker therapies.

## Author summary

Implantation of electronic pacemakers is a standard treatment to pathologically slow heart rhythm. Despite improving quality of life, those devices display many shortcomings. Bioengineered tissue pacemakers may be a therapeutic alternative, but associated design methods usually lack control of the way cells with spontaneous activity are scattered throughout the tissue. Our study is the first to use a mathematical model to rigorously define and thoroughly characterize how pacemaker cells scattering at the microscopic level may affect macroscopic behaviors of the bioengineered tissue pacemaker. Automaticity strength (ability of pacemaker cell to drive its non-pacemaker neighbors) and anisotropy (preferential orientation of cell shape) are also implemented and give unparalleled insights on how effects of uncontrollable scattered pacemaker cells may be modulated by available experimental techniques. Our model is a powerful tool to aid in optimized bioengineered pacemaker therapies.

## Introduction

Oscillating, autonomous or spontaneous electrical activity is the basis of normal heart physiology [1], as well as some impaired rhythms triggered by ectopic activity [2]. Two oscillating mechanisms or clocks, the membrane and calcium clocks, are hypothesized to control the sinoatrial node (SAN) isolated cellular rate [3–5]. Membrane clock refers to the synergy of transmembrane ionic currents [6,7], and calcium clock to the oscillations of intracellular calcium concentration [8]. Developmental variations may change magnitudes of the respective clock components [9]. Interplay between these two strongly coupled mechanisms may be responsible for spontaneous activity and temporal fluctuation in heart rate [10]. At the cellular level, the clocks basically create an ionic imbalance during the diastolic period, leading to a net inward flux of ionic current that slowly increases membrane potential until the threshold (~ −40 mV) to fire an action potential is reached. Inducing this net inward flux of ionic current during the diastole can actually generate automaticity in otherwise quiescent cardiomyocytes (CMs). This principle has been exploited in the design of biological pacemakers (BPs), a therapeutic alternative to overcome the shortcomings of cardiac electronic pacemakers [11] in the treatment of bradycardia. Different procedures have been proposed, including injection-based gene [12] and cell therapy [13], that locally modify cardiomyocyte phenotype or bring differentiated cells in the myocardium.

These concepts are limited by the lack of control on the spatial distribution and phenotype of pacemaker (PM) cells within the resting but excitable cellular network of the myocardium. We have shown that density and spatial distribution of PM cells can alter significantly the emergence and characteristics of multicellular spontaneous activity [14]. In fact, density and spatial distribution of PM cells, *a priori* unknown in BPs, may lead to a non-negligible intrinsic variability in the spontaneous activity of the overall network. Intrinsic variability is defined as behavioral discrepancies among BP samples that had undergone the exact same protocol. This phenomenon could eventually compromise the success of BP implantation in patients, and is observed even in *in vitro* BP models like monolayer cultures of neonatal rat ventricular myocytes (NRVMs), which are also heterogeneous network of autonomous and quiescent cardiomyocytes [15].

In the present simulation study, besides density and spatial distribution of PM cells, we introduce two additional variables: (a) automaticity strength and (b) anisotropy. Automaticity strength is defined as the ability of a pacemaker cell to maintain robust spontaneous activity with fast rate and to drive neighboring quiescent cells. It is strongly related to the amplitude of the net inward ionic current into the PM cell during the late diastolic period and the rising phase of the action potential (AP). For example, adding fetal bovine serum to monolayer cultures of NRVMs “strengthens” automaticity, i.e. favors higher firing rate, by upregulating inward long-lasting activation calcium current I_CaL_ [16]. Early versions of engineered BPs have been created from quiescent monolayer cultures or quiescent *in vivo* CMs via the use of different techniques to upregulate inward pacemaking current I_f_ [17]. The second newly introduced variable, anisotropy, can be created in cultures of NRVMs via several methods, notably by patterning the culture substrate [18,19] or directly seeding the cell into a thin slice of decellularized cardiac tissue [20]. These methods usually lead to functional cardiac network with elongated cell and faster propagation in the longitudinal direction [20]. It has been proposed that linear anisotropy could facilitate BP function [21]. However, the underlying mechanism remains unclear since most studies do not assess specific effects of anisotropy on spontaneous activity but instead focus on contractile function [20], electrical activation [22], or orientation-related response to stretch [23].

This study aims to assess modulation effects of automaticity strength and anisotropy on the spontaneous activity of cardiac monolayers with various densities and spatial distributions. The non-linear relationship between those two variables and automaticity will be characterized with simulation methods and discussed in details.

## Methods

### Cardiac network model

Semi-discrete microstructure models are more suitable than continuous models when individual cell sizes, shapes and orientations are variables under investigation [24,25]. For this reason, a previously described semi-discrete microstructure model [26] was used to simulate two 2D network geometries corresponding to isotropic and anisotropic monolayers. The two network geometries were identical in all aspects, except: (a) aspect ratio of cells (AR, length divided by width of the cell), and (b) distribution of gap junctions. As summarized in Table 1, a grid of 920 x 920 nodes was created with 6 μm resolution, and assigned to 42,642 CMs to create a 5.5 mm x 5.5 mm monolayer.

**Table 1:** Characterization of the monolayers: isotropic vs. anisotropic. Isotropic and anisotropic monolayers are identical for most of the features. The cells differ only in aspect ratio and intercellular conductivities.

	Isotropic	Anisotropic
Total number of nodes (#)	920 x 920	920 x 920
Resolution (μm)	6	6
Length of monolayer (mm)	5.5	5.5
Width of monolayer (mm)	5.5	5.5
Area of monolayer (mm^2^)	30.5	30.5
Total number of cells (#)	42642	42642
Number of nodes per cell (# nodes)	20 ± 4	20 ± 3
Number of neighbors (#)	6 ± 1	6 ± 1
Aspect ratio of cell (n.u.)	1.02 ± 0.26	2.92 ± 0.62
Length of cells (μm)	31.4 ± 5.3	53.6 ± 6.5
Width of cells (μm)	31.5 ± 5.2	18.9 ± 3.0
Intracellular resistivity (Ω cm)	200	200
Longitudinal intercellular conductivity (nS)	0.04	0.062
Transverse intercellular conductivity (nS)	0.04	0.034
Longitudinal conduction velocity (cm/s)	15.0	24.3
Transverse conduction velocity (cm/s)	15.2	10.4

Each cell included ~20 nodes. CMs for anisotropic geometry had an average AR of 3 compared to 1 for isotropic geometry. Longitudinal and transverse intercellular conductivities were adjusted to fit experimental conduction velocities found in NRVMs monolayer cultures [18]. The experimental isotropic conduction velocity was reported to be 16.8 ± 2.1 cm/s in all directions; and for anisotropic monolayer cultures, the longitudinal and transverse conduction velocities were 20.8 ± 3.2 cm/s and 10.9 ± 2.9 cm/s respectively. Intercellular coupling was set to 0.04 nS per 6 μm border length for CMs in isotropic network, and 0.062 nS and 0.034 nS per 6 μm border length respectively along longitudinal and transverse borders of CMs in anisotropic network.

**Fig 1:**
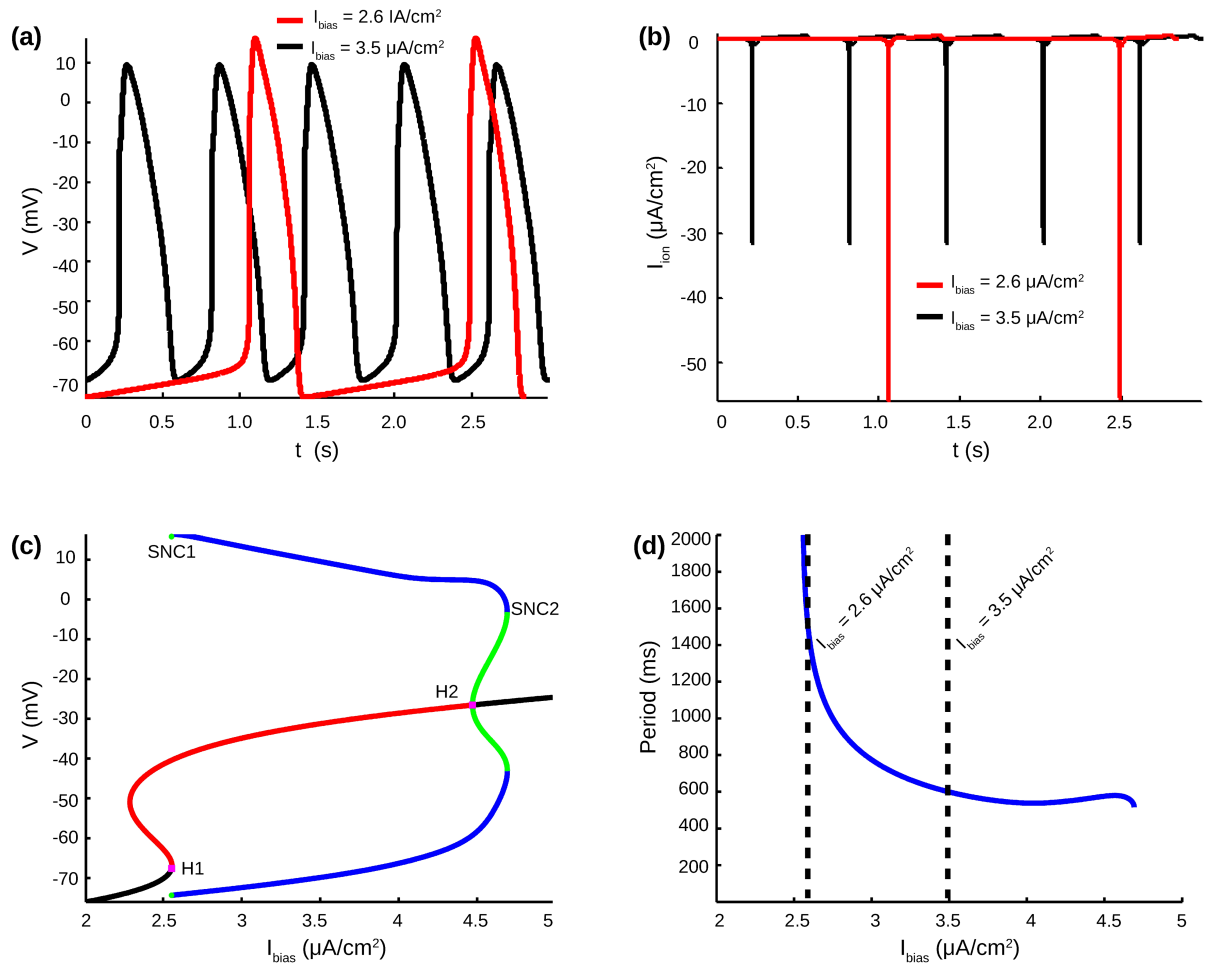
Cardiac monolayer model. (**a**) Example of spontaneous APs obtained for *I*_*bias*_ = 2.6 μA/cm^2^ and *I*_*bias*_ = 3.5 μA/cm^2^. (**b**) Total ionic currents corresponding to AP traces in panel a. (**c**) Stable and unstable fixed points (black and red line respectively), with subcritical Hopf bifurcations H1 and H2 (magenta squares, *I*_*bias*_ = 2.554 and 4.470 μA/cm^2^). Maximum and minimum membrane potential V values of the stable and unstable cycles (blue and green lines respectively). The stable cycles exists between the two cycle saddle nodes bifurcation SNC1 and SNC2 (*I*_*bias*_ = 2.553 and 4.691 μA/cm^2^). (**d**) Autonomous cycle lengths as a function of I_bias_ (stable cycles only). Dashed lines display cycle length for I_bias_ = 2.6 μA/cm^2^ and I_bias_ = 3.5 μA/cm^2^, corresponding to AP traces in panel a.

The Luo-Rudy Phase 1 (LR1) mathematical model of ventricular cell [27] was used to represent the CMs, with the application of a constant inward bias current (*I*_*bias*_) to generate spontaneous activity [28,29]. The LR1 model is simulated at every node of each cell, and examples of APs and total ionic currents obtained in a single cell with *I*_*bias*_ = 2.6 μA/cm^2^ and *I*_*bias*_ = 3.5 μA/cm^2^ are illustrated in Fig 1A and 1B respectively.

This cell model was chosen because its bifurcation structure related to oscillatory behavior has been fully characterized. Indeed, bifurcation analysis undertaken with AUTO continuation software [30] is displayed in Fig 1C. The S-shape curve of fixed points has a lower and upper branch connected by an intermediate branch of unstable fixed points. Both the lower and upper branches change stability through subcritical Hopf Bifurcations (H1 and H2, magenta square). Stable cycle exist in between the two Cycle Saddle nodes bifurcation (blue lines from SNC1 and SNC2). At high *I*_*bias*_, the branch of unstable cycles created at SNC2 (green line) connect the Hopf bifurcation H2. The branch of unstable cycles created at SNC2 exist only on a small interval of *I*_*bias*_ and ends through a Homoclinic bifurcation with the intermediate branch of fixed point. Similarly, the branch of unstable cycles created at H1 exist on a tiny interval of *I*_*bias*_ and also disappears through a Homoclinic bifurcation with the intermediate branch of fixed point. The cycle length of stable spontaneous activity decreased with *I*_*bias*_ and ranged from 1989 ms to 516 ms (Fig 1D).

### Stochastic distribution of pacemaker cells

**Fig 2:**
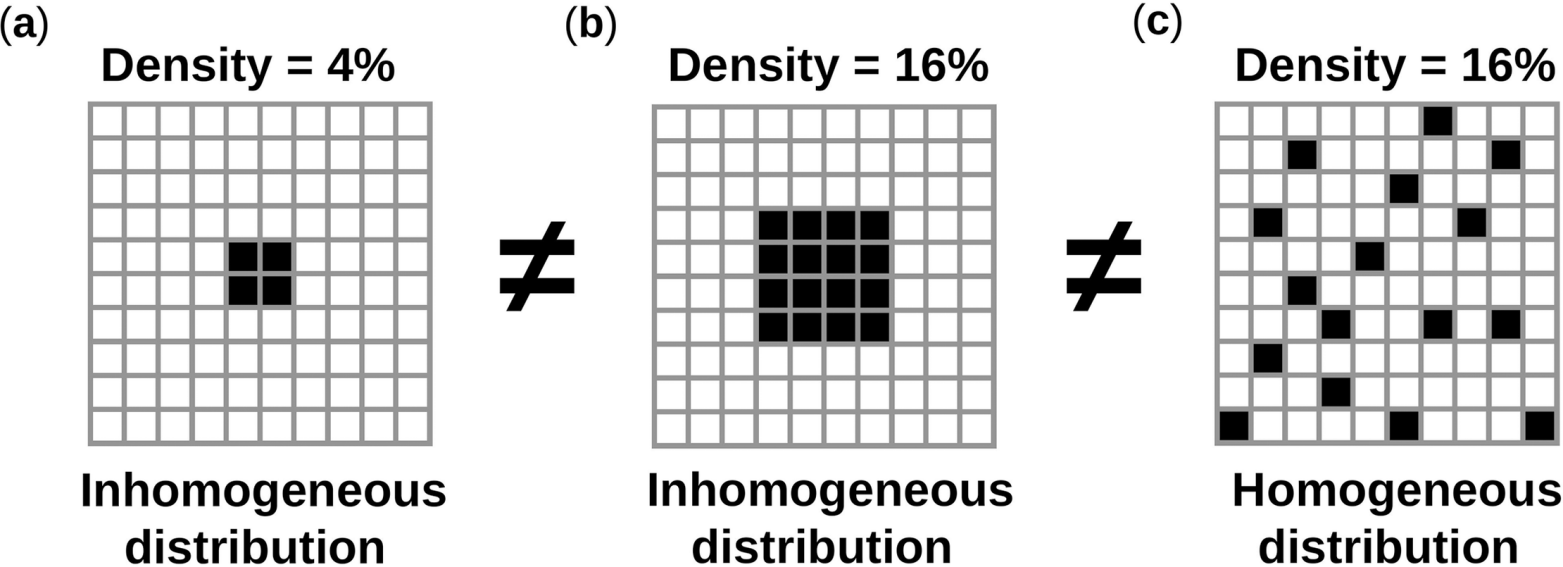
Disambiguation: density vs. spatial distribution. (**a**) Density of 4% with inhomogeneous distribution. (**b**) Density of 16% with inhomogeneous distribution. (**c**) Density of 16% with homogeneous distribution.

The 2D cardiac network was assumed to contain two populations of cells: PM (*I*_*bias*_ = 2.6 μA/cm^2^ or *I*_*bias*_ = 3.5 μA/cm^2^) and quiescent (*I*_*bias*_ = 0 μA/cm^2^) excitable cells. The density of pacemaker cells (D_aut_ ∈ [0,1]) was defined as the percentage of PM cells within the network. The spatial distribution was dependent on a variable (*p*_*thr*_ ∈ [0,1]) determining how homogeneous PM cells were spread in the network. Fig 2 provides intuitive disambiguation between density and spatial distribution. Density described the total number of PM cells in the network, regardless of their scattering within the network.

**Fig 3:**
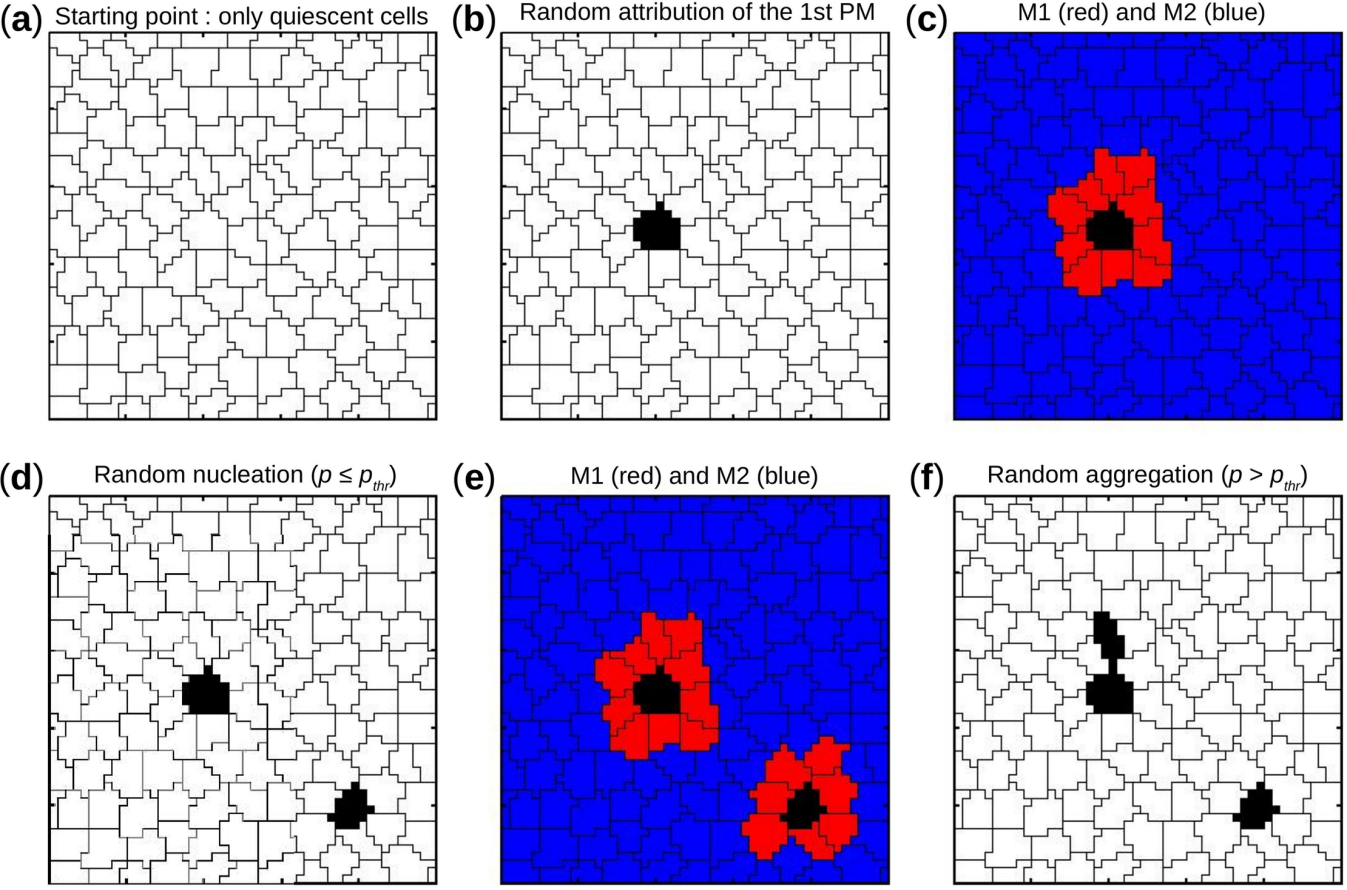
Stochastic algorithm governing density and spatial distribution of pacemaker cells: An illustration. (**a**) Blank geometry where all cells are quiescent. (**b**) Random attribution of the 1^st^ pacemaker cell. (**c**) Determination of M_1_ available sites for aggregation and M_2_ available sites for nucleation in red and blue respectively. (**d**) Random nucleation of the 2^nd^ pacemaker cell in M_2_ eventually because *p* ≤ *p*_*thr*_. (**e**) Determination of M_1_ and M_2_. (**f**) Random aggregation of the 3^rd^ pacemaker cell in M_1_ eventually because *p* > *p*_*thr*_.

Spatial distribution described the scattering of PM cells, and hence completed the spatial information from the overall density. Both variables were required to fully specify a spatial pattern. Two networks might have the same density of PM cells but different spatial distributions, or conversely they might share similar spatial distribution of PM cells but had different densities. Higher values of D_aut_ and *p*_*thr*_ correlate with higher percentage and more homogeneous spatial distribution of PM cells respectively. A previously described stochastic algorithm [14] was modified and implemented to randomly attribute positions to PM cells in the isotropic and anisotropic networks. Aggregation occurred when a PM cell was placed in the immediate neighborhood of another PM cell. Otherwise nucleation was said to occur, i.e. the PM cell occupied a position where only quiescent CMs were in its immediate neighborhood. Any random process of the algorithm followed continuous uniform probability distribution. For each pair (D_aut_, *p*_*thr*_), the algorithm worked as stated below and as illustrated in Fig 3:

***Step 1*:** randomly place the 1^st^ PM cell in the network (equal probability for each cell of the network);
***Step 2*:** determine M_1_ (aggregation available sites) and M_2_ (nucleation available sites);
***Step 3*:** randomly generate decision number *p* ∈ [0,1];
***Step 4*:** perform aggregation in M_1_ (if *p* > *p*_*thr*_) or nucleation in M_2_ (if *p* ≤ *p*_*thr*_);
***Step 5*:** perform aggregation in M_1_ (if M_2_ is empty);
***Step 6*:** repeat steps 2 to 5 until the required D_aut_ is reached.

Default *I*_*bias*_ value for all cells was 0, i.e. all cells are quiescent unless set otherwise. Placing a PM cell consisted in setting *I*_*bias*_ for all nodes of a cell to a single non-zero value inside the interval [2.6–4.7] μA/cm^2^. The same procedure was used to distribute spontaneous cells in the isotropic and anisotropic cell layouts.

**Fig 4:**
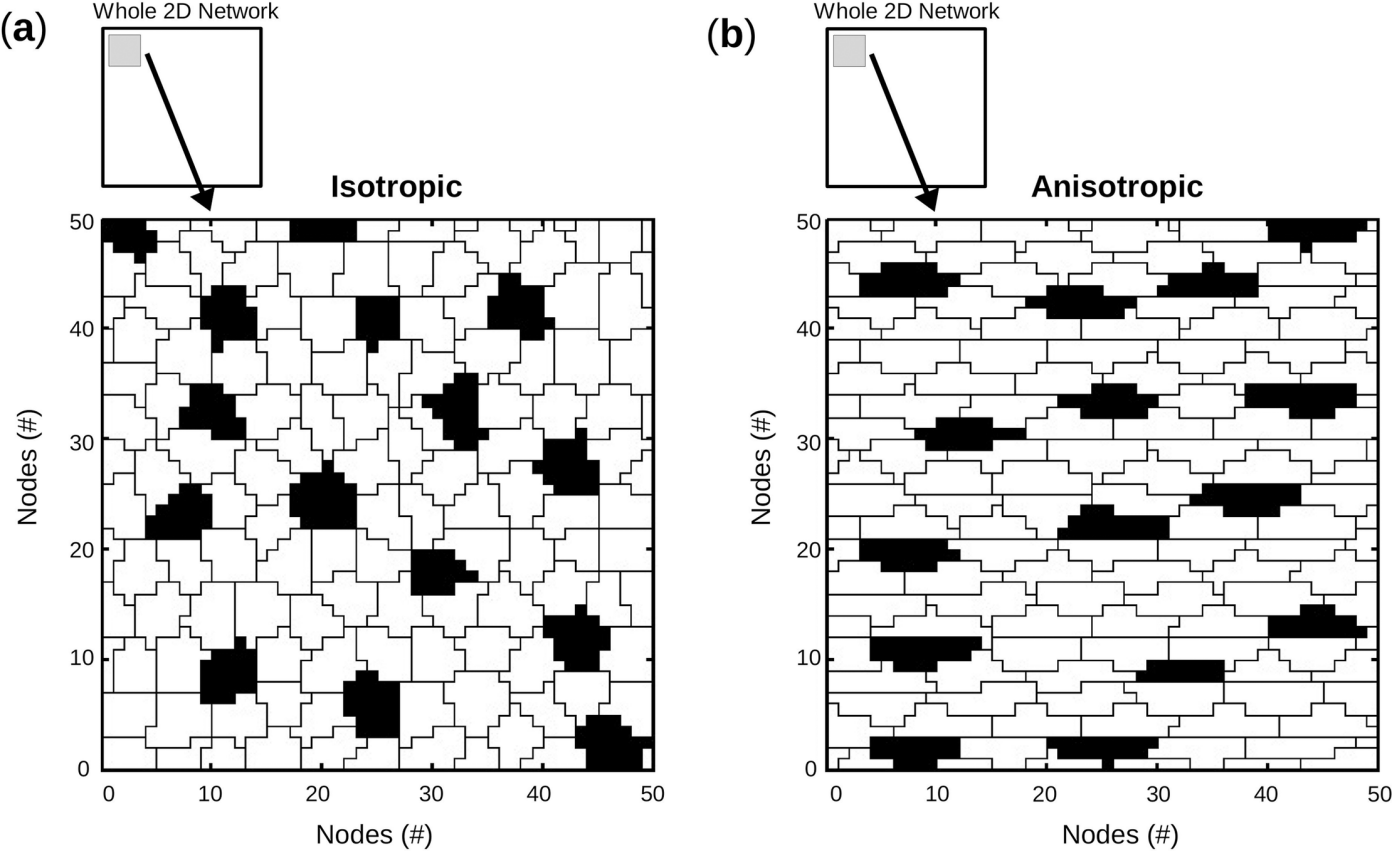
Network geometry: Isotropic vs. anisotropic. Illustration of the 50 x 50 first nodes of 2 monolayers, with pacemaker cells in black and quiescent cells in white. (**a**) Isotropic monolayer where cells display no preferential orientation. (**b**) Anisotropic monolayer where cells display preferential orientation along the longitudinal/horizontal axis.

In Fig 4 are presented the first 50 x 50 nodes of two geometries. Cells in network with isotropic geometry demonstrated no preferential orientation compared to cells in network with anisotropic geometry which are clearly oriented along the longitudinal axis.

Within the cardiac 2D network, a PM cluster was a subgroup of interconnected PM cells. Two PM cells were considered interconnected if they shared gap junctions. The size of a PM cluster was the number of PM cells in that cluster, and the maximum PM cluster size S_cluster_ was the size of the network’s biggest PM cluster. Porosity was the fraction of quiescent cells in a PM cluster. In fact, any subgroup of interconnected PM cells was a PM cluster, but quiescent cells might also be enclosed within the cluster, i.e. totally surrounded by the cluster’s PM cells. Given ${\bar{S}}_{\mathrm{Tcluster}}$, the average of maximum PM cluster size including both PM and quiescent cells, porosity was defined as follows:

$$Porosity=1-\frac{{\bar{S}}_{cluster}}{{\bar{S}}_{Tcluster}}$$

where

*Porosity*: average fraction of quiescent cells in the largest PM cluster

${\bar{S}}_{\mathrm{cluster}}$: average of maximum PM cluster size, counting only PM cells

${\bar{S}}_{\mathrm{Tcluster}}$: average of maximum PM cluster size, counting both PM and quiescent cells

### Simulation protocols and data analysis

As previously described [26], a 2D monodomain approximation with fine discretization was used to formulate the microstructure model. No-flux boundary conditions were applied to the four sides of the network. Initial conditions for all cells corresponded to the resting state of quiescent cells. The total simulation duration was 10 s, and the steady-state behaviors were reached rapidly within two to three autonomous period for most spontaneous cases, the longest transient behaviors found at the transition between non-autonomous to autonomous multicellular activity. Analysis was done on simulations after removing the first action potential thus including the time from the 2^nd^ action potential up until 10 s. Simulations were performed to study the effect of D_aut_ and *p*_*thr*_ on the spontaneous activity of 4 groups:

* ISO-2.6 –networks with isotropic geometry and weak automaticity (*I*_*bias*_ = 2.6 μA/cm^2^ for all PM cells)
* ISO-3.5 –networks with isotropic geometry and strong automaticity (*I*_*bias*_ = 3.5 μA/cm^2^ for all PM cells)
* ANISO-2.6 –networks with anisotropic geometry and weak automaticity (*I*_*bias*_ = 2.6 μA/cm^2^ for all PM cells)
* ANISO-3.5 –networks with anisotropic geometry and strong automaticity (*I*_*bias*_ = 3.5 μA/cm^2^ for all PM cells)

400 pairs (D_aut_, *p*_*thr*_) were drawn from 20 values of D_aut_ ∈ {0.05, 0.1, …, 0.95} and 20 values of scaled $p_{thr}^{1/4}$ ∈ {0.05, 0.1, …, 1}. We used non-regular spacing for p_thr_ due to its nonlinear effect on cell distribution. Eight random realizations of the networks were generated for each pair (D_aut_,*p*_*thr*_) and for each group, generating 4 groups x 8 networks x 400 (D_aut_,*p*_*thr*_) = 12,800 simulations, as detailed below:

* ISO-2.6: 8 isotropic networks with weak automaticity × 400 (D_aut_,*p*_*thr*_) = 3,200 simulations
* ISO-3.5: 8 isotropic network with strong automaticity × 400 (D_aut_,*p*_*thr*_) = 3,200 simulations
* ANISO-2.6: 8 anisotropic network with weak automaticity × 400 (D_aut_,*p*_*thr*_) = 3,200 simulations
* ANISO-3.5: 8 anisotropic network with strong automaticity × 400 (D_aut_,*p*_*thr*_) = 3,200 simulations

Post-simulation analysis was performed in Matlab (The Mathworks, Natick, MA). The network was said to have spontaneous activity if two complete activations or more were detected during 10 s of simulation. Conversely, the simulation was labeled as non-automatic if a single AP or no activation was detected. The activation time of an action potential (AP) was defined as the time when the transmembrane voltage depolarizes beyond -40 mV. For the i^th^ AP of a given simulation, the activation map (M_tact,i_ in ms) was a matrix constructed from detected activation times for all nodes.

A first set of measures was computed for each 10 s simulation with spontaneous activity.

* Normalized activation map (M_ntact,i_ in ms) for the i^th^ AP was obtained as follows:

$$M_{ntact,i}=M_{tact,i}-\min\left[M_{tact,i}\right]$$ (1)

* Spontaneous cycle length map (ΔM_tact,i_ in ms) for the i^th^ AP was defined as:

$$\Delta M_{tact,i}=M_{tact,i}-M_{tact,i-1}$$ (2)

The spontaneous cycle length value (Δt_act,i_ in ms) for that map was:

$$\Delta t_{act,i}=\mathrm{median}\left[\Delta M_{tact,i}\right]$$ (3)

And the average spontaneous cycle length Δt_act,i_ in ms) for the series of N APs was:

$$\bar{\Delta t_{act}}=\frac{1}{N-1}\sum_{i=2}^{N}\Delta t_{act,i}$$ (4)

* The map of synchronization times (*M*_*τsync*,*i*_ in s/cm) for the i^th^ AP was the inverse of conduction velocities, calculated from the spatial gradient of *M*_*tact*,*i*_ using a previously described method [31]. It quantified activation delays between cells. If all cells were synchronized, meaning they activated at the same time, *M*_*τsync*,*i*_ would be zero for each position on the lattice. The average synchronization time for N APs was:

$$\tau_{sync}=\frac{1}{N}\sum_{i=1}^{N}\mathrm{median}\left[M_{\tau sync,i}\right]$$ (5)

A second set of measures were defined for each pair (D_aut_,*p*_*thr*_) to assess how spontaneous activity behaves for similar spatial patterns and hence the variability between realizations of the same random process of pattern generation. In fact, as previously stated, eight monolayers have been produced for every pairs (D_aut_,*p*_*thr*_). The monolayers produced with the same (D_aut_,*p*_*thr*_) had similar spatial patterns, compared to monolayers produced with different (D_aut_,*p*_*thr*_) which had dissimilar spatial patterns.

* For each pair (D_aut_,*p*_*thr*_), given n simulations (up to 8) with spontaneous activity, average and standard deviation of spontaneous cycle length for similar spatial patterns were defined as:

$${\bar{\Delta T}}_{act}=\frac{1}{n}\sum_{j=1}^{n}\bar{\Delta t_{act,j}}$$ (6)

$$\sigma_{\Delta Tact}=\sqrt{\frac{1}{n-1}\sum_{j=1}^{n}{\left(\bar{\Delta t_{act,j}}-{\bar{\Delta T}}_{act}\right)}^{2}}$$ (7)

${\bar{\Delta T}}_{\mathrm{act}}$: average cycle length over n simulations for a pair (D_aut_, *p*_*thr*_)

σ_ΔTact_: standard deviation of cycle length over n simulations for a pair (D_aut_, *p*_*thr*_)

$\bar{\Delta t_{act,j}}$: cycle length for the j^th^ simulation, as stated in Eq (4)

* For each pair (D_aut_,*p*_*thr*_), average and standard deviation of synchronization time for similar spatial patterns were defined as:

$${\bar{T}}_{sync}=\frac{1}{n}\sum_{j=1}^{n}\tau_{sync,j}$$ (8)

$$\sigma_{Tsync}=\sqrt{\frac{1}{n-1}\sum_{}^{}{\left(\tau_{sync,j}-{\bar{T}}_{sync}\right)}^{2}}$$ (9)

${\bar{T}}_{\mathrm{sync}}$: average synchronization time over n simulations for a pair (D_aut_, *p*_*thr*_)

σ_Tsync_: standard deviation of synchronization time over n simulations for a pair (D_aut_, *p*_*thr*_)

τ_sync,j_: synchronization time for the j^th^ simulation, as stated in Eq (5)

The same process is used to calculate ${\bar{T}}_{\mathrm{sync},x}$, ${\bar{T}}_{\mathrm{sync},y}$, longitudinal and transverse components of T_sync_.

The third set of measures were defined for dissimilar spatial patterns, i.e. monolayers with different values of (D_aut_,*p*_*thr*_).

* The cycle length range for dissimilar patterns was defined as the percentage variation between the minimum and maximum values of ${\bar{T}}_{\mathrm{sync}}$ over all pairs of (D_aut_,*p*_*thr*_):

$$\mathrm{Range}\left[{\bar{\Delta T}}_{act}\right]=100\frac{\max\left[{\bar{\Delta T}}_{act}\right]-\min\left[{\bar{\Delta T}}_{act}\right]}{\min\left[{\bar{\Delta T}}_{act}\right]}$$ (10)

${\bar{\Delta T}}_{\mathrm{act}}$: average cycle length over n simulations for a pair (D_aut_, *p*_*thr*_), as stated in Eq (6)

* The synchronization time range for dissimilar patterns was defined as the percentage variation between the minimum and maximum values of ${\bar{T}}_{\mathrm{sync}}$ over all pairs of (D_aut_,*p*_*thr*_):

$$\mathrm{Range}\left[{\bar{T}}_{sync}\right]=100\frac{\max\left[{\bar{T}}_{sync}\right]-\min\left[{\bar{T}}_{sync}\right]}{\min\left[{\bar{T}}_{sync}\right]}$$ (11)

${\bar{T}}_{\mathrm{sync}}$: average synchronization time over n simulations for a pair (D_aut_, *p*_*thr*_), as stated in Eq (8)

Furthermore, each monolayer was divided in two areas separated by a square of 2.75 mm side (half of the side of the monolayer) to distinguish between border and central foci (first initiation site of AP). Border foci behavior was assessed by anisotropy ratio. The border foci anisotropy ratio (r) was defined as:

$$r=\frac{\eta_{L}}{\eta_{T}}$$ (12)

η_L_: number of border foci in longitudinal x-direction (anisotropy direction)

η_T_: number of border foci in transverse y-direction

## Results

### Characterization of the cardiac 2D network

**Fig 5:**
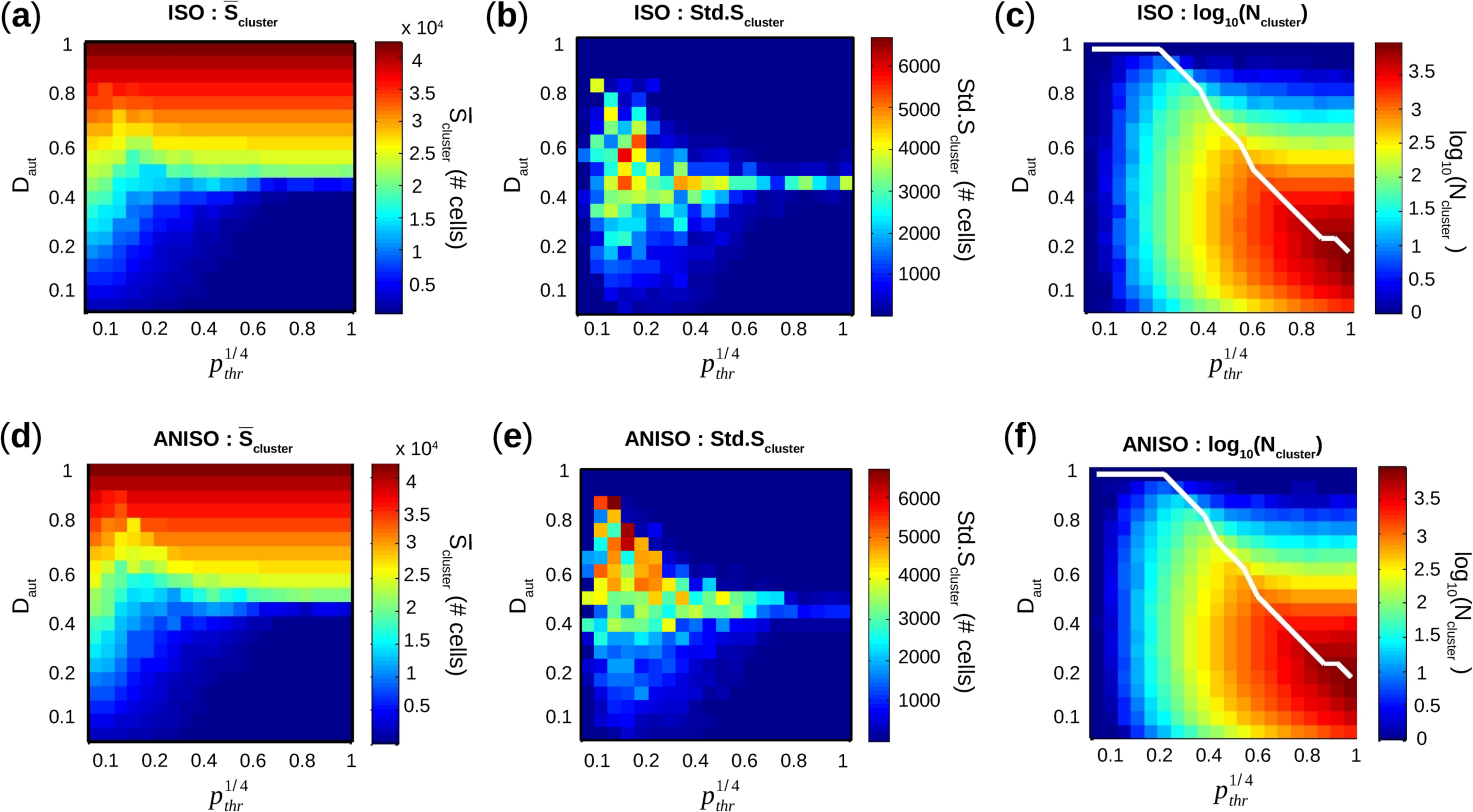
Characterization of the stochastic algorithm governing density and spatial distribution of pacemaker cells. (**a**,**d**) Average of maximum cluster size S_cluster_ in color scale map as a function of D_aut_ and $p_{thr}^{1/4}$, for monolayers with isotropic and anisotropic geometries. The size of a cluster is the actual number of pacemaker cells in that cluster. (**b**,**e**) Standard deviation of S_cluster_ vs. D_aut_ and $p_{thr}^{1/4}$. (**c**,**f**) Log_10_ of number of clusters N_cluster_ vs. D_aut_ and $p_{thr}^{1/4}$. Solid white line is D_aut,max_ (see definition in text) as a function of $p_{thr}^{1/4}$.

${\bar{S}}_{\mathrm{cluster}}$, average of S_cluster_ over 8 monolayers for each pair (D_aut_,*p*_*thr*_), increased with D_aut_, independently of $p_{thr}^{1/4}$, with a transition from below 10,000 PM cells to over 30,000 PM cells around D_aut_ = 0.5 (Fig 5A and 5D).

**Fig 6:**
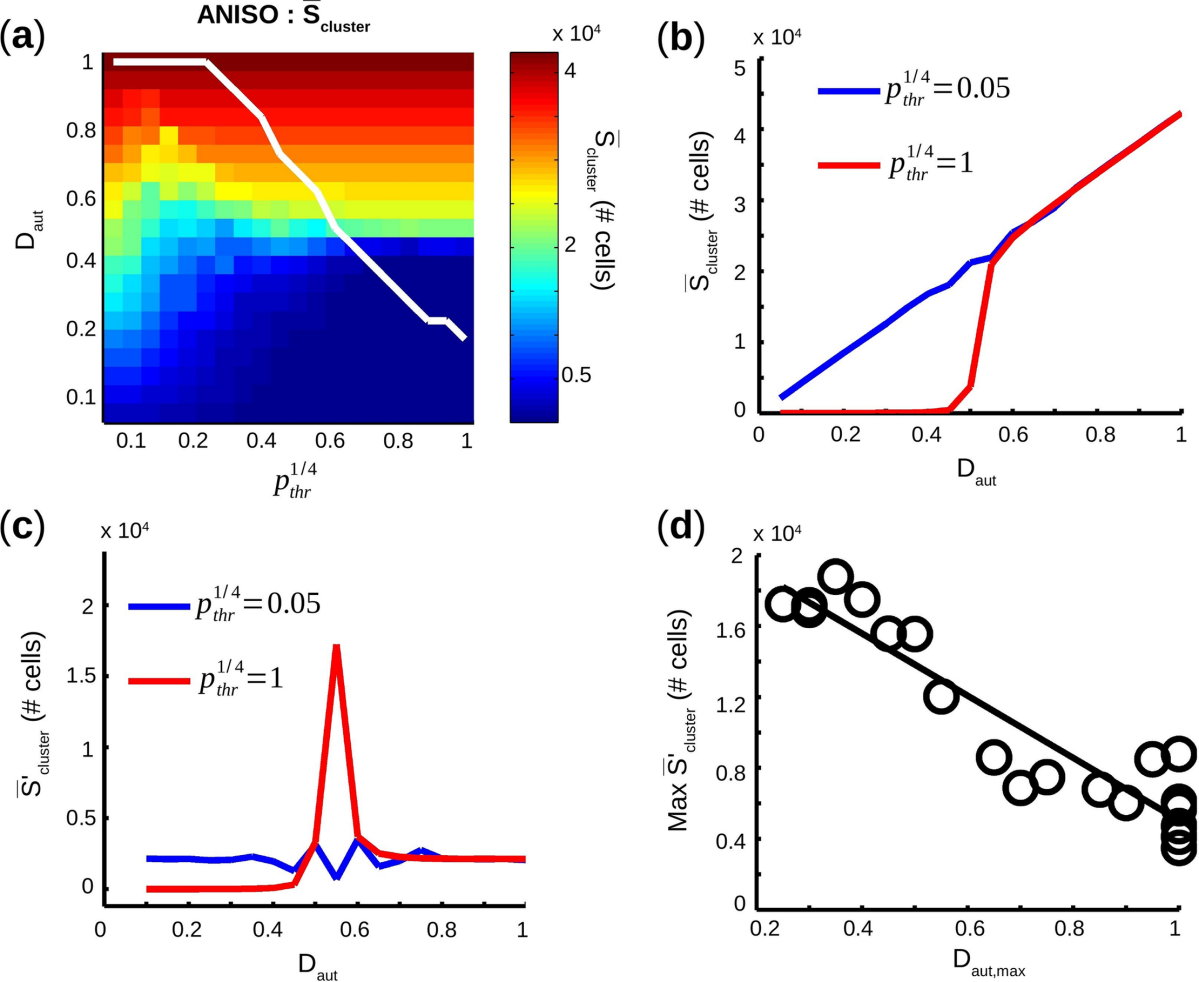
Relationship between cluster size transition and cluster fusion. (**a**) ${\bar{S}}_{\mathrm{cluster}}$ (average of maximum cluster size) in color scale map as a function a function of D_aut_ and $p_{thr}^{1/4}$, for anisotropic networks. Solid white line is D_aut,max_ as a function of $p_{thr}^{1/4}$. (**b**) ${\bar{S}}_{\mathrm{cluster}}$ as a function of D_aut_, for first and last $p_{thr}^{1/4}$. (**c**) S¯'cluster (first derivative of ${\bar{S}}_{\mathrm{cluster}}$) as a function of D_aut_, for first and last $p_{thr}^{1/4}$. (**d**) Maximum of S¯'cluster vs. D_aut_ and for all $p_{thr}^{1/4}$, plotted as a function D_aut,max_.

The extent of the transition phase, i.e. the number of pairs (D_aut_,*p*_*thr*_) with ${\bar{S}}_{\mathrm{cluster}}$ between 10,000 and 30,000 PM cells, correlated with increased standard deviation of ${\bar{S}}_{\mathrm{cluster}}$ (Std.S_cluster_). In fact, Std.S_cluster_ (Fig 5B and 5E) was below 1,000 PM cells for all pairs (D_aut_,*p*_*thr*_) except for the transition phase where Std.S_cluster_ > 2,000 PM cells. As shown in Fig 5C and 5F, the number of clusters (N_clusters_) increased with D_aut_ as long as D_aut_ ≤ D_aut,max_ (white solid line), and then decreased for D_aut_ > D_aut,max_. For each $p_{thr}^{1/4}$, D_aut,max_ was the maximum D_aut_ beyond which M_2_ became empty, i.e. there was no more available site to perform cluster nucleation during the creation of the spatial distribution of PM cells. Thus, once D_aut,max_ has been reached, only aggregation was possible for increasing D_aut_. In Fig 6, a relationship was established between the transition in S_cluster_ and D_aut,max_.

The maximum of the derivative of ${\bar{S}}_{\mathrm{cluster}}$ as a function of D_aut_, i.e. max[${\bar{S}}_{\mathrm{cluster}}^{\prime }$] = max[ΔS_cluster_/ΔD_aut_], representing the sharpness of the transition of S_cluster_ from below 10,000 PM cells to over 30,000 PM cells, was calculated for all $p_{thr}^{1/4}$ and plotted against D_aut,max_. The sharpness of the transition was inversely proportional to D_aut,max_ (Fig 6D). Differences between isotropic and anisotropic geometries were unsurprisingly negligible for ${\bar{S}}_{\mathrm{cluster}}$, N_clusters_, and D_aut,max_, since the number of neighbors was approximately the same for both geometries. As such, spatial distribution of spontaneous cells created by our aggregation and nucleation process are not affected by cell preferential orientation.

### Occurrence of spontaneous activity

**Fig 7:**
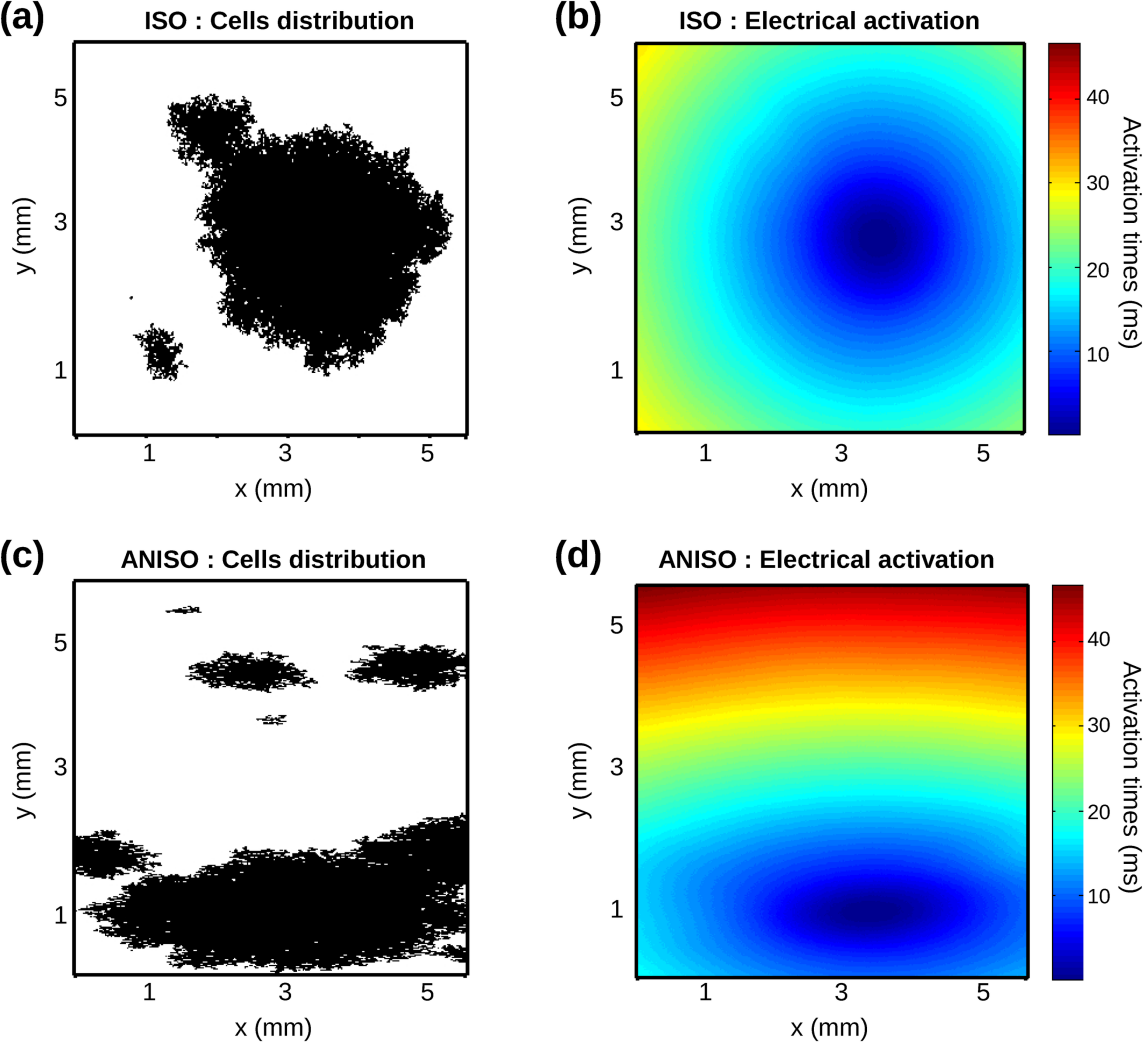
Electrical activation: isotropic vs. anisotropic. (**a**) Isotropic monolayer with D_aut_ = 0.3 and $p_{thr}^{1/4}$ = 0.10 (black sites: PM cells). (**b**) Electrical activation times (ms) color scale map as a function of node positions for the previously described isotropic monolayer. (**c**) Anisotropic monolayer with D_aut_ = 0.3 and $p_{thr}^{1/4}$ = 0.15. (**d**) Electrical activation times (ms) for the anisotropic monolayer.

In general, 2D cardiac networks with isotropic geometry demonstrated circular-shaped electrical activation, as illustrated in Fig 7B for the pattern shown in panel a. Networks with the anisotropic geometry typically had ellipse-shape electrical activation (Fig 7D for the pattern in panel c).

**Fig 8:**
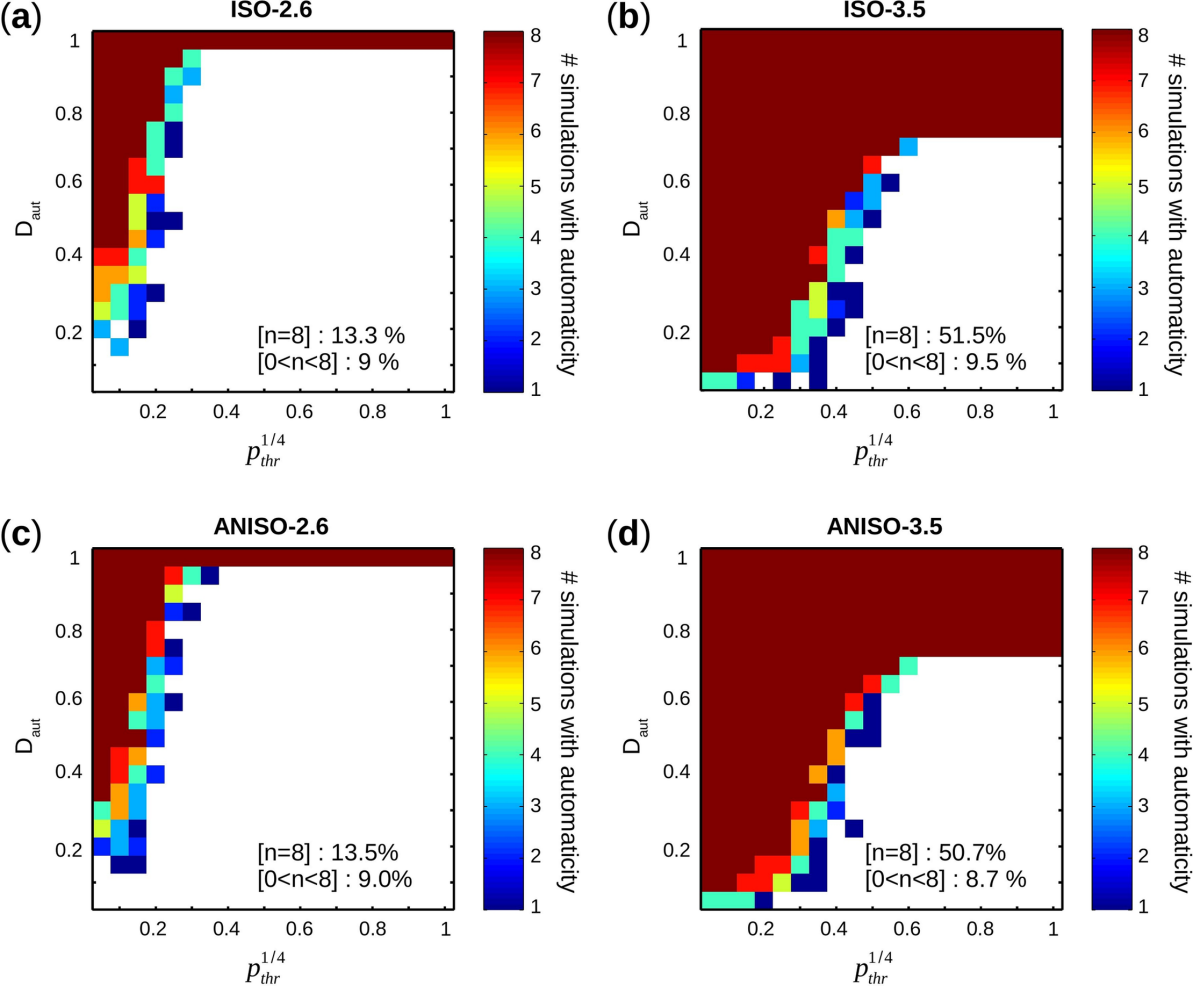
Occurrence of automaticity. (**a**-**d**) For each group, number n of simulations with automaticity is displayed in color scale map as a function D_aut_ and $p_{thr}^{1/4}$. White spots correspond to [n = 0]. Proportions of pairs (D_aut_,*p*_*thr*_) with [n = 8] and [0<n<8] are also indicated.

For a specific pair (D_aut_, *p*_*thr*_), given n the number of simulations with automaticity: (a) [n = 8] meant automaticity occurred for all 8 simulations, (b) [0<n<8] for 1 to 7 simulations, and (c) [n = 0] for no simulation with automaticity. As shown in Fig 8, autonomous activity occurs more often in ISO-3.5 and ANISO-3.5 compared to ISO-2.6 and ANISO-2.6 over all pairs (D_aut_, *p*_*thr*_).

For example, 51.5% of pairs (D_aut_,*p*_*thr*_) demonstrated [n = 8] in ISO-3.5 versus 13.3% in ISO-2.6. Interestingly, proportions of pairs (D_aut_,*p*_*thr*_) with [0<n<8] were very similar for all four groups (~9%). Automaticity was thus more likely to be observed for higher values of D_aut_ and lower values of $p_{thr}^{1/4}$, and no difference in occurrence of autonomous activity was found between isotropic and anisotropic geometries.

**Fig 9:**
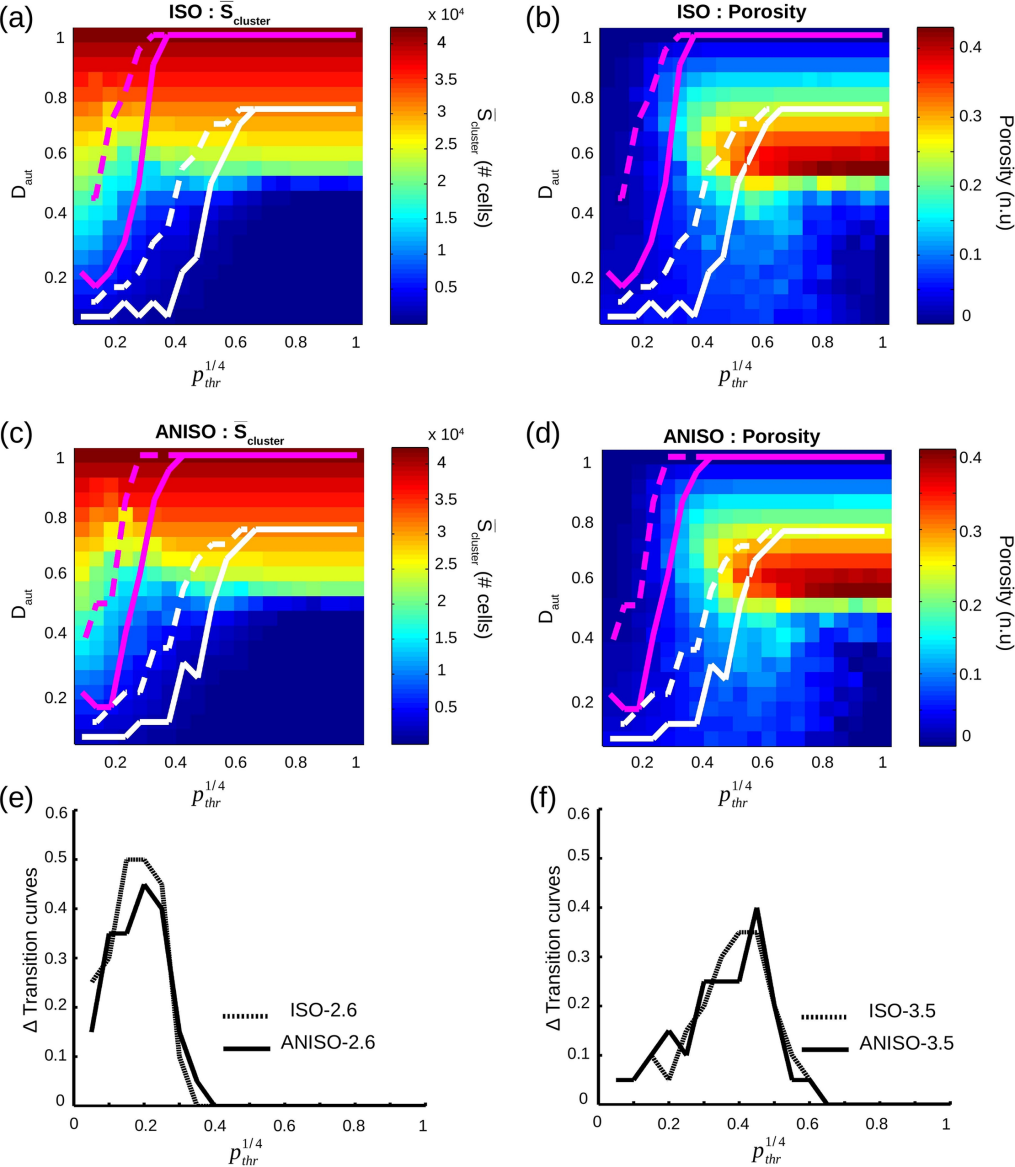
Transition curves. (**a**-**d**) Transition curves from [n = 0] to [0<n<8] (solid line) and from [0<n<8] to [n = 8] (dashed line) are displayed in magenta for ISO-2.6 and ANISO-2.6, and in white for ISO-3.5 and ANISO-3.5. In background is color scale map of either ${\bar{S}}_{\mathrm{cluster}}$ or porosity, both against D_aut_ and $p_{thr}^{1/4}$. (**e**,**f**) For each group, transition curve to [0<n<8] is subtracted to transition curve to [n = 8].

A transition curve from [n = 0] to [0<n<8] was the line drawn by the minimum D_aut_ required for each $p_{thr}^{1/4}$ to transition from [n = 0] to [0<n<8]. Similarly, the transition curve from [0<n<8] to [n = 8] was the line drawn by the minimum D_aut_ required for each $p_{thr}^{1/4}$ to transition from [0<n<8] to [n = 8]. In Fig 9A–9D, transition curves from [n = 0] to [0<n<8] (solid line) and from [0<n<8] to [n = 8] (dashed line) were superimposed to the color scale map of S_cluster_ and porosity, for isotropic and anisotropic networks.

${\bar{S}}_{\mathrm{cluster}}$ combined with porosity offered crucial insights on the morphology of the transition curves. Typically, automaticity did not appear when ${\bar{S}}_{\mathrm{cluster}}$ was below 5,000 PM cells and when porosity was over 0.35. The higher $p_{thr}^{1/4}$ was, the greater S_cluster_ had to be to generate automaticity. In all groups, larger ${\bar{S}}_{\mathrm{cluster}}$ was required to reach [n = 8] versus [0<n<8]. Networks with strong automaticity displayed spontaneous activity at smaller values of ${\bar{S}}_{\mathrm{cluster}}$ and higher porosity.

For all 4 groups, transition curves to [0<n<8] were subtracted from transition curves to [n = 8], and the differences were displayed in Fig 9E and 9F. Peak differences were higher for ISO-2.6 (0.50) and ANISO-2.6 (0.45) compared to ISO-3.5 (0.35) and ANISO-3.5 (0.40) respectively. Furthermore peak differences occurred for lower $p_{thr}^{1/4}$ for ISO-2.6 and ANISO-2.6 (~0.2) compared to ISO-3.5 and ANISO-3.5 (~0.40).

### Rate of spontaneous activity

**Fig 10:**
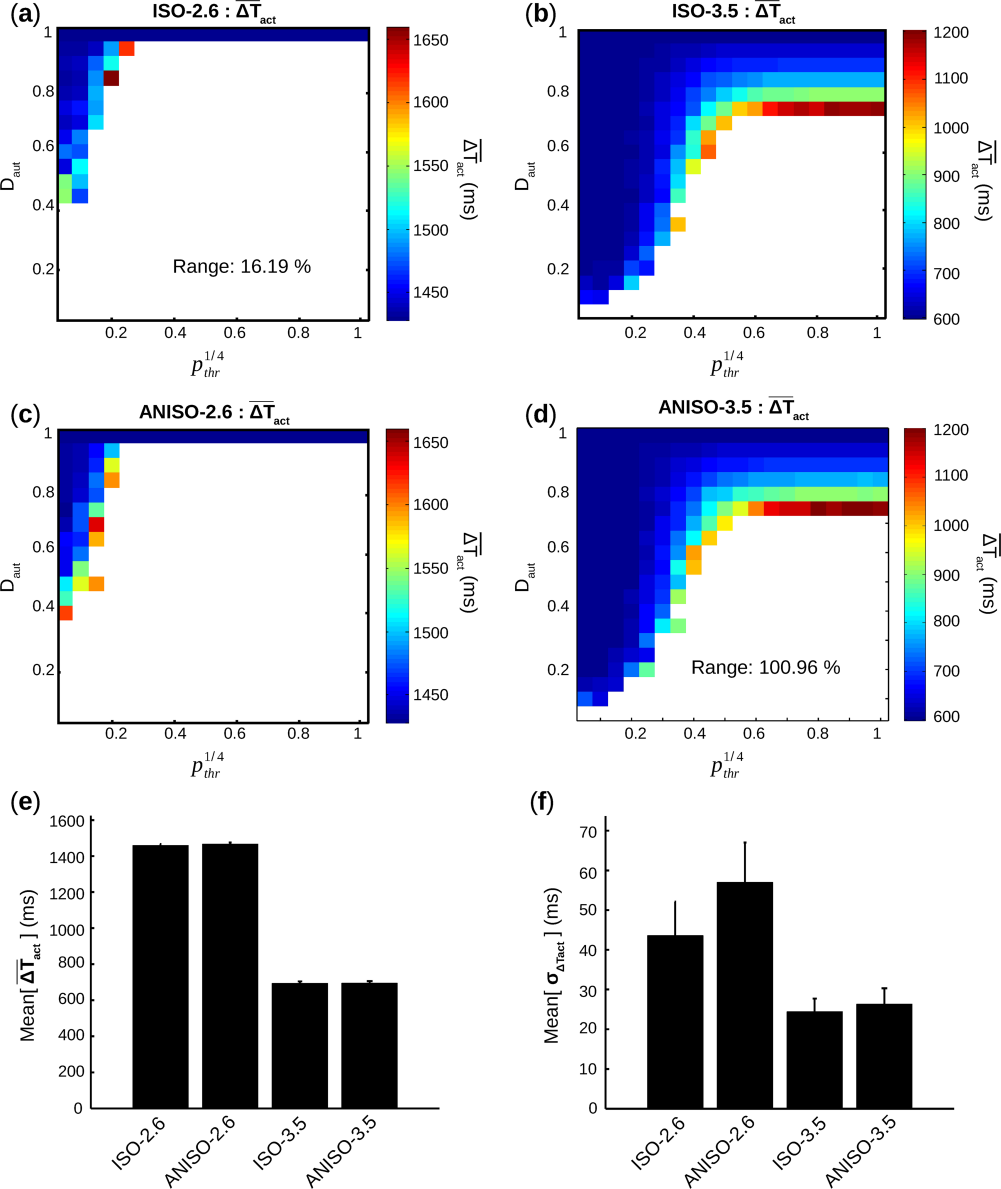
Rate of spontaneous activity. (**a**-**d**) For all groups, average cycle length ${\bar{\Delta T}}_{\mathrm{act}}$ is calculated for each pair (D_aut_,*p*_*thr*_) with [n = 8] and displayed as a color scale map. The corresponding percentage range between the minimum and the maximum values of the ${\bar{\Delta T}}_{\mathrm{act}}$ map is also displayed. (**e**) For each group, mean and s.e.m of values in ${\bar{\Delta T}}_{\mathrm{act}}$ map are calculated. (**f**) For each group, mean and s.e.m of values in Std cycle length σ_ΔTact_ map (obtained following the same process than ${\bar{\Delta T}}_{\mathrm{act}}$) are calculated.

In Fig 10A–10D, ${\bar{\Delta T}}_{\mathrm{act}}$ was calculated for each pair (D_aut_,*p*_*thr*_) with [n = 8] and displayed as color scale map vs. D_aut_ and $p_{thr}^{1/4}$.

Independently of groups and for any given $p_{thr}^{1/4}$, ${\bar{\Delta T}}_{\mathrm{act}}$ decreased with increasing D_aut_. And ranges, i.e. intrinsic variability for dissimilar patterns, of ${\bar{\Delta T}}_{\mathrm{act}}$ for ISO-2.6 (16.19%) and ANISO-2.6 (14.56%) were much smaller than ISO-3.5 (98.86%) and ANISO-3.5 (100.96%).

In Fig 10E is presented mean[${\bar{\Delta T}}_{\mathrm{act}}$], calculated as the average ${\bar{\Delta T}}_{\mathrm{act}}$ over all pairs (D_aut_,*p*_*thr*_) with [n = 8] shown in Fig 10A–10D. Detailed values can be found in Table 2.

**Table 2:** Summary of simulation results.

	ISO 2.6	ANISO 2.6	ISO 3.5	ANISO 3.5
Range[${\bar{\Delta T}}_{\mathrm{act}}$] (%) [n = 8]	16.2	14.6	98.9	101.0
Mean[${\bar{\Delta T}}_{\mathrm{act}}$] (ms) [n = 8]	1460 ± 6	1467 ± 8	694 ± 10	696 ± 10
Mean[**σ**_**ΔTact**_] (ms) [n = 8]	44 ± 9	57 ± 10	24 ± 3	26 ± 4
Mean[${\bar{\Delta T}}_{\mathrm{act}}$] (ms) [0<n<8]	1702± 25	1754± 28	1078± 39	1137± 49
Mean[**σ**_**ΔTact**_] (ms) [0<n<8]	120± 10	175± 21	193± 27	208± 19
Central foci (%) [n = 8]	8.33	2.57	21.7	12.8
Central foci (%) [0<n<8]	1.42	0.775	16.2	12.6
r [n = 8]	1.28	1.62	0.93	2.82
r [0<n<8]	1.26	5	1	2.19
Range[${\bar{T}}_{\mathrm{Sync}}$] (%) [n = 8]	6.5	16.9	414.0	484.7
Mean[${\bar{T}}_{\mathrm{Sync}}$] (s/cm) [n = 8]	0.0665 ± 2×10^−4^	0.0876 ± 6×10^−4^	0.0640 ± 6×10^−4^	0.0811 ± 10×10^−4^
Mean[**σ**_**TSync**_] (s/cm) [n = 8]	0.00094 ± 2×10^−4^	0.0047 ± 3×10^−4^	0.0020 ± 2×10^−4^	0.0064 ± 3 ×10^−4^
Mean [${\bar{T}}_{\mathrm{sync},x}$] (s/cm) [n = 8]	0.0436 ± 7×10^−4^	0.0131 ± 9×10^−4^	0.0404 ± 6×10^−4^	0.0160 ± 3×10^−4^
Mean [${\bar{T}}_{\mathrm{sync},y}$**]** (s/cm) [n = 8]	0.0433 ± 7×10^−4^	0.0857 ± 8×10^−4^	0.0418 ± 6×10^−4^	0.0781 ± 10×10^−4^

Mean[${\bar{\Delta T}}_{\mathrm{act}}$] for ISO-2.6 and ANISO-2.6 were respectively 110.26% and 110.96% higher compared to those of ISO-3.5 and ANISO-3.5. This behavior was not surprising, considering the difference observed in single cell simulations (1428 ms for *I*_*bias*_ = 2.6 μA/cm^2^ compared to 599 ms for *I*_*bias*_ = 3.5 μA/cm^2^, in Fig 1D). Besides, it is important to notice that, for a given *I*_*bias*_ value, single pacemaker cells always display lower cycle length than monolayers. No difference in mean[${\bar{\Delta T}}_{\mathrm{act}}$] was found between isotropic and anisotropic geometries for [n = 8], but focusing on the set with [0<n<8] yielded interesting results. Pooling together all pairs (D_aut_, *p*_*thr*_) satisfying the condition [0<n<8] (Table 2), there was a higher mean[${\bar{\Delta T}}_{\mathrm{act}}$] ANISO-2.6 versus ISO-2.6 (3.08%), and ANISO-3.5 versus ISO-3.5 (5.50%).

Similarly to ${\bar{\Delta T}}_{\mathrm{act}}$, σ_ΔTact_ was calculated for each pair (D_aut_,*p*_*thr*_) with [n = 8]. Mean[σ_ΔTact_], the average value of σ_ΔTact_ over all pairs (D_aut_,*p*_*thr*_) with [n = 8] is also shown in Fig 10F and Table 2 for all groups. Mean[σ_ΔTact_] was a measure of intrinsic variability between monolayers with similar spatial patterns of PM cells. Mean[σ_ΔTact_] for ISO-2.6 and ANISO-2.6 were respectively 78.52% and 116.68% higher compared ISO-3.5 and ANISO-3.5. A difference between isotropic and anisotropic geometries was found where mean[σ_ΔTact_] in ANISO-2.6 was 30% higher vs. ISO-2.6, while a more limited increase of 8% was found for ANISO-3.5 compared to ISO-3.5.

### Spatial characteristics of spontaneous activity

**Fig 11:**
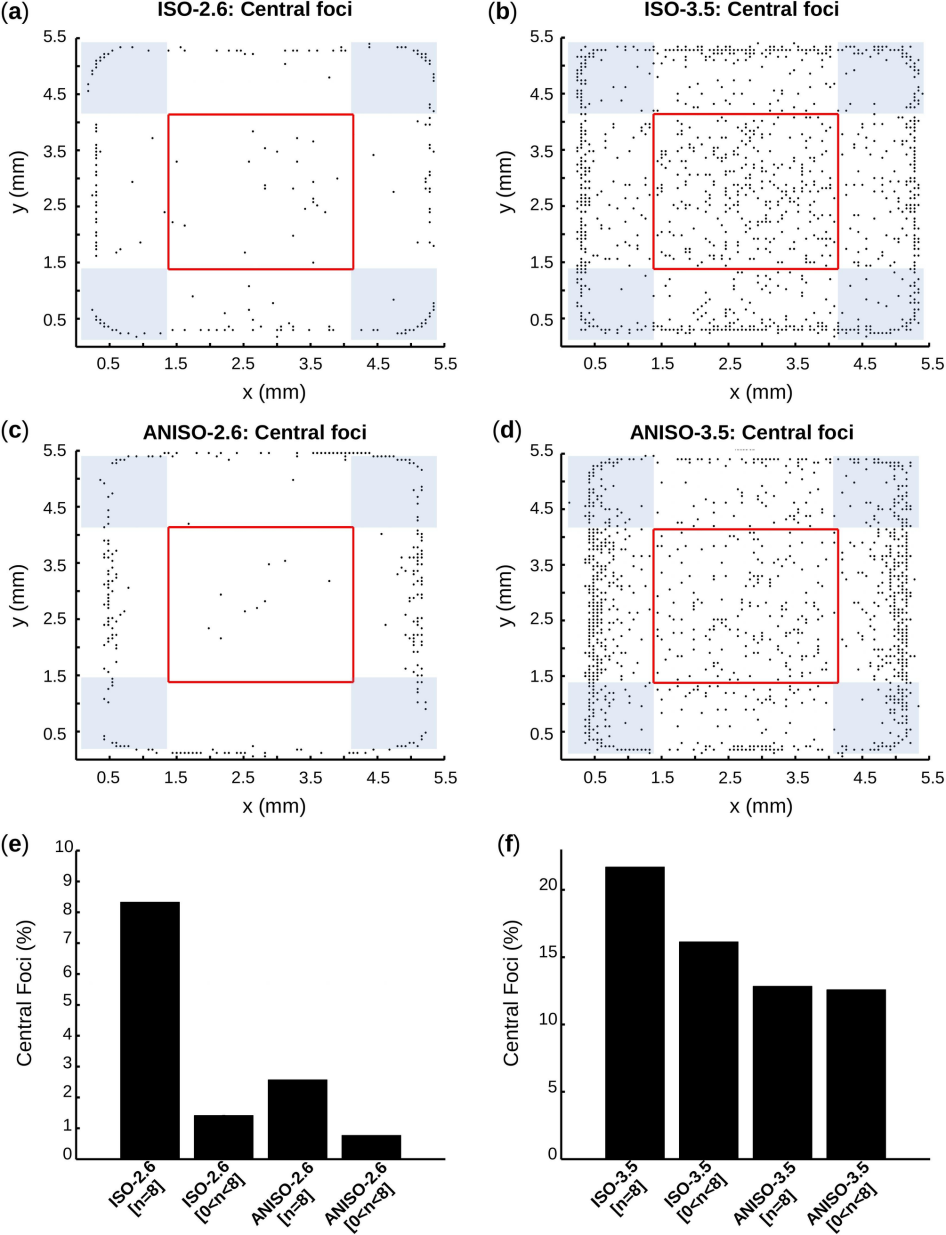
Foci positions: Central vs. border. (**a**-**d**) For each group, and for [n>0], focal position of the last activation is plotted. Central foci are inside the red square, whose side is 50% of the monolayer side. The border foci in the longitudinal x-direction are the foci located outside the red box, exclusively to the left and to the right. The border foci in the transverse y-direction are exclusively at the top and the bottom. Non-exclusive border foci at the corners, i.e. foci that are common to longitudinal and transverse direction are in the blue areas and are not considered in the calculation of border foci anisotropy ratio (r) in Eq (12). (**e**,**f**) Proportions of central focals for [n = 8] and [0<n<8].

Difference in spatial characteristics of spontaneous activity between isotropic and anisotropic geometry was evaluated. The position of foci (i.e. first initiation sites of electrical activation) was estimated to determine if differences existed between the two network geometries. Independently of geometry, focal activation was highly stable in time for a given spatial pattern of spontaneous cells, demonstrating no beat-to-beat variability. For direct comparison between geometries, the focal position of the last simulated spontaneous beat was selected for all simulations with automaticity (i.e. [n>0]). Pooled positions were plotted in Fig 11A–11D.

The proportion of central foci over all foci is shown in Fig 11E and 11F and Table 2 for pairs of (D_aut_,*p*_*thr*_) with [n = 8] and pairs (D_aut_,*p*_*thr*_) with [0<n<8]. Anisotropic geometries demonstrated fewer central foci, independently of *I*_*bias*_ or n values. For pairs (D_aut_,*p*_*thr*_) with [n = 8], proportions of central foci decreased by 69.11% from ISO-2.6 to ANISO-2.6, and by 40.84% from ISO-3.5 to ANISO-3.5. The drop was less important for pairs of (D_aut_,*p*_*thr*_) with [0<n<8] case, where proportions of central foci fell by 45.35% from ISO-2.6 to ANISO-2.6, and by 22.05% from ISO-3.5 to ANISO-3.5. It was also interesting to observe that cases with [0<n<8] demonstrated fewer central foci than cases with [n = 8], independently of *I*_*bias*_ value or geometry. In fact, in ISO-2.6 and ANISO-2.6, proportions of central foci respectively fell by 83% and 69.9% from [n = 8] to [0<n<8]. The difference between pairs of (D_aut_,*p*_*thr*_) with [n = 8] and [0<n<8] was less important when *I*_*bias*_ = 3.5 μA/cm^2^. As a matter of fact, the drop of central foci proportions from [n = 8] to [0<n<8] was 25.58% in ISO-3.5 and 1.94% in ANISO-3.5.

The border foci in the longitudinal x-direction were the foci located outside the red box, exclusively to the left and to the right. The border foci in the transverse y-direction were the foci located outside the red box, exclusively at the top and the bottom. Non-exclusive border foci at the corners, i.e. foci that are common to longitudinal and transverse directions (blue areas in Fig 11A–11D), were not considered in the calculations. Ratio with values greater than one suggested that there were more border foci in the longitudinal direction compared to the transverse direction. Values of *r* were calculated for [n = 8] and [0<n<8] and are presented in Table 2. Interestingly, *r* in anisotropic geometry are consistently greater than one and always higher compared to the value obtained in isotropic geometry. In fact, for [n = 8] case, *r* raised by 27% from ISO-2.6 to ANISO-2.6, and by 204% from ISO-3.5 to ANISO-3.5. For [0<n<8], *r* raised by 297% from ISO-2.6 to ANISO-2.6, and by 119% from ISO-3.5 to ANISO-3.5. No clear preference in border foci position was found for the isotropic network.

**Fig 12:**
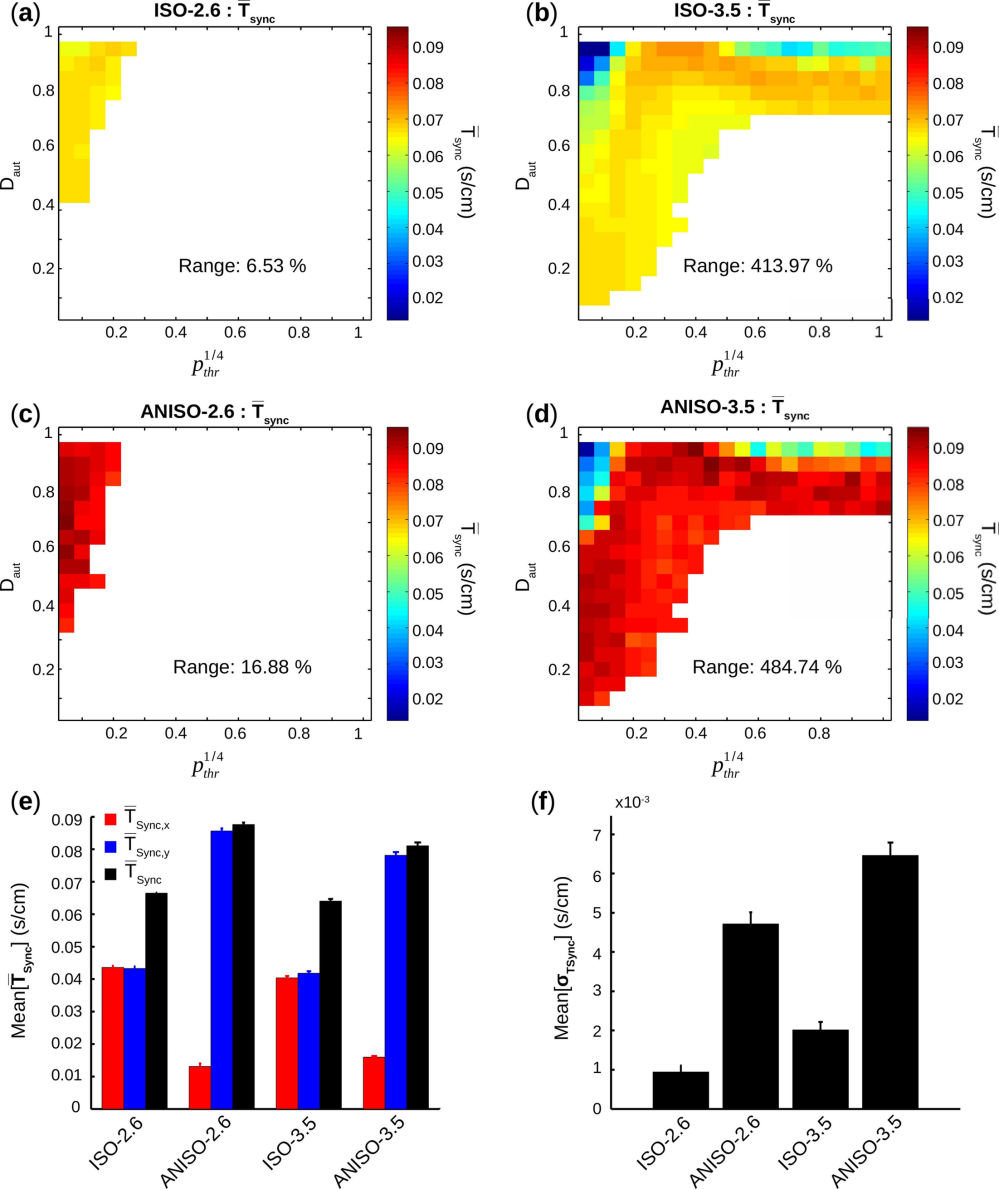
Synchronization times. (**a**-**d**) For all groups, average synchronization time ${\bar{T}}_{\mathrm{sync}}$ is calculated for each pair (D_aut_,*p*_*thr*_) with [n = 8] and displayed as a color scale map. The corresponding percentage range between the minimum and the maximum values of the ${\bar{T}}_{\mathrm{sync}}$ map is also displayed.(**e**) For each group, mean and s.e.m of values in ${\bar{T}}_{\mathrm{sync},x}$, ${\bar{T}}_{\mathrm{sync},y}$, ${\bar{T}}_{\mathrm{sync}}$ map are calculated. (**f**) For each group, mean and s.e.m of values in Std synchronization time σ_TSync_ map (obtained following the same process than ${\bar{T}}_{\mathrm{sync}}$) are calculated.

Synchronization of electrical activation is an important marker of spontaneously beating multicellular monolayer, either *in silico* or *in vitro*. ${\bar{T}}_{\mathrm{sync}}$ was displayed as color map vs. D_aut_ and $p_{thr}^{1/4}$ for each pair (D_aut_,*p*_*thr*_) with [n = 8] in Fig 12A–12D.

No particular tendencies were observed in ${\bar{T}}_{\mathrm{sync}}$ map for ISO-2.6 and ANISO-2.6. For ISO-3.5 and ANISO-3.5, and for D_aut_ ≥ 0.8, ${\bar{T}}_{\mathrm{sync}}$ increased and then decreased as a function of $p_{thr}^{1/4}$. *I*_*bias*_ = 3.5 μA/cm^2^ led to much higher ranges compared to *I*_*bias*_ = 2.6 μA/cm^2^. Indeed, range[${\bar{T}}_{\mathrm{sync}}$] for ISO-2.6 (6.5%) and ANISO-2.6 (16.9%) were much smaller than ISO-3.5 (414%) and ANISO-3.5 (485%). Moreover, the increase in ranges could be noticed in anisotropic geometries versus isotropic geometries: 160% increase from ISO-2.6 to ANISO-2.6 and more moderate 17.15% increase from ISO-3.5 to ANISO-3.5.

Anisotropy consistently yielded higher synchronization times. Mean[${\bar{T}}_{\mathrm{sync}}$] rose by 31.87% from ISO-2.6 to ANISO-2.6 and by 26.64% from ISO-3.5 to ANISO-3.5. Mean[${\bar{T}}_{\mathrm{sync},y}$] obviously played an important role in that increase. In fact, the unsurprising decrease of synchronization times in the direction of anisotropy mean[${\bar{T}}_{\mathrm{sync},x}$] was associated with a dramatic increase of mean[${\bar{T}}_{\mathrm{sync},y}$], leading to a higher resultant mean[${\bar{T}}_{\mathrm{sync}}$].

## Discussion

To our knowledge, this paper presents the first *in silico* study in a microstructure model describing how automaticity strength and structural linear anisotropy may modulate the effects of density and spatial distribution of PM cells on the spontaneous activity of the BP.

Our previous study [14] demonstrated that not only the density but also the spatial distribution of PM cells may induce important intrinsic variability in the BP dynamical behavior. The study focused on a simple continuous and isotropic 2D substrate with a single period of activity for the autonomous cells. These results quickly raised a very important question: can this intrinsic variability be limited despite the lack of control on density and spatial distribution of autonomous cells? Two straightforward solutions may be considered: (a) either the BP is tested ex vivo and then implanted into the myocardium, or (b) the current methods are combined with new processes intended to minimize the intrinsic variability. Investigating the second candidate solution is the main motivation behind the mathematical modeling optimization process that is underway. Here, we studied how different automaticity strengths and structural linear anisotropy can influence the spatial-temporal activity of the BP.

The main new contributions are described as follows:

1. Spatial patterns of PM cells were mathematically defined and characterized within a semi-discrete 2D model, describing both automaticity strength and structural linear anisotropy.
2. Automaticity strength enhanced occurrence of spontaneous activity, decreased variability in cycle length ${\bar{\Delta T}}_{\mathrm{act}}$ and synchronization time ${\bar{T}}_{\mathrm{sync}}$ for similar spatial patterns of PM cells, but increased the number of central foci, and the variability of ${\bar{\Delta T}}_{\mathrm{act}}$ and ${\bar{T}}_{\mathrm{sync}}$ for dissimilar spatial patterns PM cells.
3. Structural linear anisotropy had no important effect on occurrence of spontaneous activity, increased variability in ${\bar{\Delta T}}_{\mathrm{act}}$ and ${\bar{T}}_{\mathrm{sync}}$ for both similar and dissimilar spatial patterns of PM cells, and decreased the proportion of central foci.
4. Intrinsic variability was modulated but not eliminated by neither automaticity strength nor structural linear anisotropy, since there was still a proportion of pairs (D_aut_,*p*_*thr*_) with no spontaneous activity and important performance discrepancies notably for dissimilar spatial patterns of PM cells.

PM cells were randomly placed in a semi-discrete 2D microstructure via a stochastic algorithm with a parameter D_aut_ controlling density and a parameter $p_{thr}^{1/4}$ determining homogeneity of spatial distribution. Two levels of automaticity strength (weak and strong) were achieved by two different values of *I*_*bias*_ (2.6 μA/cm^2^ and 3.5 μA/cm^2^). Linear anisotropy was structurally created by: (1) geometrically increasing aspect ratio of cells from one to three, and (2) fitting longitudinal and transverse gap junction distribution to published conduction velocities from anisotropic monolayer cultures of NRVMs [18].

Isolated PM cell dynamics were described by LR1 cardiac ventricle myocyte model [27], with a constant inward bias current to generate automaticity, as described elsewhere [28,29]. More complex ionic models (for example the published NRVM ionic model [32] or a mathematical models representing stem cell-derived human cardiomyocytes [33]) were not used: (1) to avoid important computational cost because of the associated long transient dynamics and (2) because increasing dimensions of mathematical ionic models does not necessarily lead to more predictive power. As a matter of fact, in many high dimension models very different set of parameters can lead to the same AP; in this case the model is said to have identifiability issue, a major flaw to predictive reliability [34]. The LR1 model obviously does not include the detailed ionic currents of cardiomyocytes (pacemaking and resting) nor can it reproduce the intricate interaction between the membrane/voltage clocks [6,28]. However it remains an appropriate trade-off option since it is identifiable [34] and can reproduce the basic physiological behaviors considered in the present study, namely automaticity and AP propagation.

To avoid confounding effects, some simplifications were made:

1. We considered only two populations of cells: PM and quiescent excitable cells. Other types of heterogeneities such as sinks (spots with voltage fixed at resting potential like fibroblasts) and breaks (spots with no conduction, like monolayer damage) were not considered although they may have had an effect on conduction [35].
2. *I*_*bias*_ was the same for all PM cells of a specific monolayers, i.e. all PM cells were identical. All cells had the same initial conditions and no time delay. It is important to notice however that clinical BPs with all identical PM cells would lack robustness to external perturbations. For example they may completely stop firing after acetylcholine stimulation. Cellular diversity is indeed an important aspect to preserve robustness in the native sinus node [36].
3. The number of neighbors was approximately the same for each cell. Different number of neighbors could create electrotonic disparities between PM clusters and induce confusion with effects of porosity on occurrence of automaticity.

No reentry or asynchronous activation was detected in the simulations; this fact is an indication of strong intercellular coupling [28,29,37–39] but also may be limited by the homogeneous initial conditions used in the simulations. But the strongest coupling is not necessarily the best for clinical *in situ* BPs, without no-flux boundary conditions. In fact, to maintain source-sink balance with the atrium, native SAN cells are coupled with low conductance connexins (Cx-45) instead of high conductance Cx-43 [40]. There is indeed an inverse proportional relationship between coupling strength and the safety factor of propagation [41].

${\bar{S}}_{\mathrm{cluster}}$ and N_cluster_ as a function of D_aut_ and $p_{thr}^{1/4}$ displayed remarkable similarities with our previous study [14], despite different model structure and different number of neighbors. In fact, average of the maximum PM cluster size ${\bar{S}}_{\mathrm{cluster}}$ monotonically increased with D_aut_, with a transition around D_aut_ = 0.5 (Fig 5). However, in this study we brought deeper spatial characterizations compared to the previous one. For example, the sharpness of this transition was demonstrated to be inversely proportional to D_aut,max_ (Fig 6). The sharpness of the transition relied on two related phenomena: aggregation of clusters at low $p_{thr}^{1/4}$ and «fusion» [14] of clusters at high $p_{thr}^{1/4}$. For low $p_{thr}^{1/4}$ (heterogeneous spatial distribution of PM cells) S_cluster_ grew mainly because of aggregation and was proportional to D_aut_ which increased linearly, hence showing a smooth transition. For higher $p_{thr}^{1/4}$ (homogeneous spatial distribution) S_cluster_ grew mainly because of «fusion» since there was more nucleation and less aggregation. In fact, as D_aut_ increases, clusters start having common neighboring available sites. When a PM cell is placed on one of those sites, previously separated clusters «fuse» into one bigger cluster. Decrease of N_cluster_ indicates a start in the «fusion» process, systematically above D_aut,max_. So ${\bar{S}}_{\mathrm{cluster}}$ started growing slowly for high $p_{thr}^{1/4}$ and low D_aut_ because there was almost no aggregation and no «fusion». Once D_aut,max_ was reached, «fusion» process provoked a sharp increase of ${\bar{S}}_{\mathrm{cluster}}$ and hence a sharp transition.

Higher D_aut_ and lower $p_{thr}^{1/4}$ led to more chance of having automaticity ([n>0]) (**Fig 8**). Even higher D_aut_ and lower $p_{thr}^{1/4}$ were necessary for all simulations to show automaticity ([n = 8]). The lower $p_{thr}^{1/4}$ was, the lower D_aut_ was required to generate automaticity.

The transition curves are also a major characterization improvement, compared to our previous study [14]. Superimposing transition curves over ${\bar{S}}_{\mathrm{cluster}}$ and porosity color scale maps (Fig 9) gave crucial information about relationship between the monolayer microstructure organization and occurrence of automaticity. It was really interesting to observe that small ${\bar{S}}_{\mathrm{cluster}}$ at low $p_{thr}^{1/4}$ could generate spontaneous activity while large ${\bar{S}}_{\mathrm{cluster}}$ at high $p_{thr}^{1/4}$ could not. A big cluster at high $p_{thr}^{1/4}$ may contain too much inside spots with quiescent cells and not be able to fire an AP, while a small cluster at low $p_{thr}^{1/4}$ containing no inside quiescent spot at all will be able to do it. This is where porosity remarkably came into play. Porosity established the importance of electrotonic source-sink balance inside the PM cluster itself.

Spontaneous activity occurs more often with strong automaticity (*I*_*bias*_ = 3.5 μA/cm^2^). Basically, lower ${\bar{S}}_{\mathrm{cluster}}$ was needed and higher porosity was tolerated for automaticity with stronger PM cells to be observed.

For each $p_{thr}^{1/4}$ value, average autonomous cycle length ${\bar{\Delta T}}_{\mathrm{act}}$ decreased with increasing D_aut_ (Fig 10) since bigger clusters (with reasonably low porosity) have more electrotonic driving force. Unsurprisingly, strong automaticity brought smaller period of activity (lower ${\bar{\Delta T}}_{\mathrm{act}}$), but also resulted in higher ranges of ${\bar{\Delta T}}_{\mathrm{act}}$, i.e. more intrinsic variability for dissimilar spatial patterns. Stronger automaticity allowed occurrence of spontaneous activity in more difficult conditions (lower ${\bar{S}}_{\mathrm{cluster}}$, and higher porosity), but the resulting periods would be noticeably higher compared to more favorable conditions. On a clinical point of view, this result means that creating BPs with stronger automaticity would not solve the problem of not having control over the fate of the PM cells *in situ* (intrinsic variability). The generated BP may have better chance to display automaticity, but would have very disparate performances from one patient to another. The best way to bypass the problem is to create multiple BPs *ex vivo*, pick the cultured BP with the targeted performance and then graft it to the patient myocardium.

In accordance with the literature [14,42,43], foci were usually located at the border (Fig 11) since, with no-flux boundary condition, border clusters had less cells to depolarize. Strong PM cells led to more central foci because more clusters had enough electrotonic driving force to depolarize neighboring cells without interacting with the no-flux boundary condition. It was interesting to notice that anisotropy yielded more border foci in the longitudinal direction compared to the transverse direction. In fact, because of the aspect ratio, there were actually more cells at the two longitudinal borders and thus more chance of having a sufficiently bigger ${\bar{S}}_{\mathrm{cluster}}$ to fire an AP.

Electrical activations were less synchronous in anisotropic monolayers (Fig 12). Very low ${\bar{T}}_{\mathrm{sync}}$ in the longitudinal direction were hindered by very high ${\bar{T}}_{\mathrm{sync}}$ in the transverse direction, leading to an electrical activation that was globally less synchronous. The evolution of ${\bar{T}}_{\mathrm{sync}}$ as a function of $p_{thr}^{1/4}$ for D_aut_ ≥ 0.8 is very interesting and requires further investigations. Further investigations are also needed to explain how structural linear anisotropy consistently induced more intrinsic variability for both similar and dissimilar spatial patterns. Anisotropy had also limited effect on occurrence of automaticity and ${\bar{\Delta T}}_{\mathrm{act}}$, compared to strength of automaticity. If there was indeed an effect, it eventually happened below the 5% resolution of D_aut_ and $p_{thr}^{1/4}$. Automaticity strength and anisotropy may not be as independent as displayed in this study. Anisotropy has been shown to induce changes in intracellular calcium transients ([Ca^2+^]_i_) dynamics, decreasing the diastolic [Ca^2+^]_i_ levels and increasing [Ca^2+^]_i_ influx per cardiac cycle [44]. Anisotropy may also alter the properties of voltage-gated ion channels, notably expression and regulatory properties of voltage-gated calcium channels [45]. So, on a strict structural point of view (i.e. aspect ratio and distribution of gap junctions), anisotropy may not have an impact, but it may indirectly affect automaticity strength, which then will have an effect on spontaneous activity. As such, even if linear structural anisotropy does not increase occurrence of automaticity and does not decrease intrinsic variability, it may still be clinically interesting.

This study offered crucial insights on the relationships between the microstructure of the BP and its macroscopic behavior. Automaticity strength and structural linear anisotropy may modulate effects of density and spatial distribution of PM cells on the spontaneous activity of the BP, but will probably not eliminate intrinsic variability among BPs. Therapeutic procedures that do not take characteristics of spatial distribution into account (eg. cell and gene therapies) may end up with a non-negligible intrinsic variability, despite standardized protocols. Increasing the number of pacemaker cells may not solve the issue. As a matter of fact, the native SAN tissue exhibits a specific architecture with gradual transition of intermediate cells and gradually decreasing density of pacemaker cells from the center to the periphery. That spatial arrangement promotes automaticity by maintaining the delicate balance in the source-sink relationship between the SAN and the surrounding atrial tissue [36]. With an unknown spatial arrangement, that balance may randomly be compromised, hence the intrinsic variability. Furthermore, unlike the SAN which is electrically isolated from the rest of the atrium with the exception of few exit pathways, BPs lack electrotonic barrier at the macroscopic level, another challenge to their performance. All those facts stress the importance of *ex vivo* design and performance assessments on BPs in bioreactors before implantation to patients.

A sheet-based BP as a replacement to the normal sinus node has important intrinsic differences with the physiological structure. The normal pacemaker is a compact node of heterogeneous spontaneous cells believed to be connected at specific exit points thus limiting contact to a restricted number of atrial cells [46]. The simulated BP in this study are very different where a population of autonomous cells are connected to resting ventricular myocytes, both having similar morphology and dimensions. Cell dimensions and morphologies vary between pacemaker cells (assuming spindle-like cell morphology as in the sinus node) and resting but excitable cardiomyocyte (with also differences between atrial and ventricular cells). Cardiomyocyte dimensions is well known to affect electrical propagation [47]. Thus, important work is still needed to study the differences in spontaneous activity of BP monolayer when considering morphological differences between cell types although the differences between cell types (sinus node cell, atrial and ventricular-like derived cardiomyocytes) for derived cardiomyocytes may not be as important as in adult hearts. More importantly, BP activity when electrically coupled to myocardium in order to drive the tissue will be depressed by the electrotonic effect [48] and biomimetism of the sinus node coupling structure to the atria may need to be considered for coupling the BP to the myocardium.

### Conclusion

In summary, a pure change from isotropic to anisotropic substrate modelled by an elongated cell shape and anisotropic intercellular conductivity without modifications of ion channel expression nor spatial distribution has limited effects on spontaneous activity. However, increasing the intrinsic rate of autonomous cells has a much stronger effects. Although the two were studied together and independently, it is of importance to note that there is a strong possibility that both changes (anisotropy and autonomous strength) could be coupled [44]. Further work is thus needed to uncover this importance of the interaction (and by how much methods to induce cellular anisotropy can increase the cellular automaticity strength) and to elucidate how it could favor the BP development.

## Supporting information

S1 MovieTime course of activation times for the isotropic geometry and weak pacemaker cells.Parameters are I_bias_ = 2.6 μA/cm^2^, D_aut_ = 0.8 and $p_{thr}^{1/4}$ = 0.1.(MP4)

S2 MovieTime course of activation times for the anisotropic geometry and weak pacemaker cells.Parameters are I_bias_ = 2.6 μA/cm^2^, D_aut_ = 0.8 and $p_{thr}^{1/4}$ = 0.1.(MP4)

S3 MovieTime course of activation times for the isotropic geometry and strong pacemaker cells.Parameters are I_bias_ = 3.5 μA/cm^2^, D_aut_ = 0.3 and $p_{thr}^{1/4}$ = 0.1.(MP4)

S4 MovieTime course of activation times for the anisotropic geometry and strong pacemaker cells.Parameters are I_bias_ = 3.5 μA/cm^2^, D_aut_ = 0.3 and $p_{thr}^{1/4}$ = 0.1.(MP4)

## References

[1] Mangoni ME, Nargeot J. Genesis and Regulation of the Heart Automaticity. Physiol Rev. 2008;88: 919–982. doi: 10.1152/physrev.00018.2007 18626064 10.1152/physrev.00018.2007

[2] Rozanski GJ, Lipsius SL. Electrophysiology of functional subsidiary pacemakers in canine right atrium. Am J Physiol. 1985;249: H594–603. doi: 10.1152/ajpheart.1985.249.3.H594 4037107 10.1152/ajpheart.1985.249.3.H594

[3] Monfredi O, Maltsev VA, Lakatta EG. Modern Concepts Concerning the Origin of the Heartbeat. Physiology. 2013;28: 74–92. doi: 10.1152/physiol.00054.2012 23455768 10.1152/physiol.00054.2012 PMC3768086

[4] Maltsev VA, Lakatta EG. Dynamic interactions of an intracellular Ca2+ clock and membrane ion channel clock underlie robust initiation and regulation of cardiac pacemaker function. Cardiovasc Res. 2008;77: 274–284. doi: 10.1093/cvr/cvm058 18006441 10.1093/cvr/cvm058

[5] Maltsev VA, Vinogradova TM, Lakatta EG. The Emergence of a General Theory of the Initiation and Strength of the Heartbeat. J Pharmacol Sci. 2006;100: 338–369. doi: 10.1254/jphs.CR0060018 16799255 10.1254/jphs.cr0060018

[6] Severi S, Fantini M, Charawi LA, DiFrancesco D. An updated computational model of rabbit sinoatrial action potential to investigate the mechanisms of heart rate modulation. J Physiol. 2012;590: 4483–4499. doi: 10.1113/jphysiol.2012.229435 22711956 10.1113/jphysiol.2012.229435 PMC3477753

[7] DiFrancesco D. The Role of the Funny Current in Pacemaker Activity. Circ Res. 2010;106: 434–446. doi: 10.1161/CIRCRESAHA.109.208041 20167941 10.1161/CIRCRESAHA.109.208041

[8] Stern MD, Maltseva LA, Juhaszova M, Sollott SJ, Lakatta EG, Maltsev VA. Hierarchical clustering of ryanodine receptors enables emergence of a calcium clock in sinoatrial node cells. J Gen Physiol. 2014;143: 577–604. doi: 10.1085/jgp.201311123 24778430 10.1085/jgp.201311123 PMC4003189

[9] Sasse P, Zhang J, Cleemann L, Morad M, Hescheler J, Fleischmann BK. Intracellular Ca2+ Oscillations, a Potential Pacemaking Mechanism in Early Embryonic Heart Cells. J Gen Physiol. 2007;130: 133–144. doi: 10.1085/jgp.200609575 17664344 10.1085/jgp.200609575 PMC2151640

[10] Zaniboni M, Cacciani F, Lux RL. Beat-to-Beat Cycle Length Variability of Spontaneously Beating Guinea Pig Sinoatrial Cells: Relative Contributions of the Membrane and Calcium Clocks. PLOS ONE. 2014;9: e100242 doi: 10.1371/journal.pone.0100242 24940609 10.1371/journal.pone.0100242 PMC4062511

[11] Munshi NV, Olson EN. Improving cardiac rhythm with a biological pacemaker. Science. 2014;345: 268–269. doi: 10.1126/science.1257976 25035474 10.1126/science.1257976 PMC4316825

[12] Hu Y-F, Dawkins JF, Cho HC, Marbán E, Cingolani E. Biological pacemaker created by minimally invasive somatic reprogramming in pigs with complete heart block. Sci Transl Med. 2014;6: 245ra94 doi: 10.1126/scitranslmed.3008681 25031269 10.1126/scitranslmed.3008681 PMC4949602

[13] Cai J, Lin G, Jiang H, Yang B, Jiang X, Yu Q, et al Transplanted neonatal cardiomyocytes as a potential biological pacemaker in pigs with complete atrioventricular block. Transplantation. 2006;81: 1022–1026. doi: 10.1097/01.tp.0000214954.09515.51 16612279 10.1097/01.tp.0000214954.09515.51

[14] Duverger JE, Boudreau-Béland J, Le MD, Comtois P. Multicellular automaticity of cardiac cell monolayers: effects of density and spatial distribution of pacemaker cells. New J Phys. 2014;16: 113046 doi: 10.1088/1367-2630/16/11/113046

[15] Guo W, Kamiya K, Cheng J, Toyama J. Changes in action potentials and ion currents in long-term cultured neonatal rat ventricular cells. Am J Physiol. 1996;271: C93–102. doi: 10.1152/ajpcell.1996.271.1.C93 8760034 10.1152/ajpcell.1996.271.1.C93

[16] Gomez JP, Potreau D, Branka JE, Raymond G. Developmental changes in Ca2+ currents from newborn rat cardiomyocytes in primary culture. Pflugers Arch. 1994;428: 241–249. 7816546 10.1007/BF00724503

[17] Boink GJJ, Christoffels VM, Robinson RB, Tan HL. The past, present, and future of pacemaker therapies. Trends Cardiovasc Med. 2015;25: 661–673. doi: 10.1016/j.tcm.2015.02.005 26001958 10.1016/j.tcm.2015.02.005

[18] Bursac N, Parker KK, Iravanian S, Tung L. Cardiomyocyte Cultures With Controlled Macroscopic Anisotropy. Circ Res. 2002;91: e45–e54. doi: 10.1161/01.RES.0000047530.88338.EB 12480825 10.1161/01.res.0000047530.88338.eb

[19] Liau B, Christoforou N, Leong K, Bursac N. Pluripotent Stem Cell-derived Cardiac Tissue Patch with Advanced Structure and Function. Biomaterials. 2011;32: 9180–9187. doi: 10.1016/j.biomaterials.2011.08.050 21906802 10.1016/j.biomaterials.2011.08.050 PMC3190071

[20] Blazeski A, Kostecki GM, Tung L. Engineered heart slices for electrophysiological and contractile studies. Biomaterials. 2015;55: 119–128. doi: 10.1016/j.biomaterials.2015.03.026 25934457 10.1016/j.biomaterials.2015.03.026 PMC4780840

[21] Campbell PH, Feinberg AW, Goss JA, Parker KK, Ripplinger CM. Anisotropic biological pacemakers and av bypasses [Internet]. WO2012048242 A1, 2012. Available: http://www.google.ca/patents/WO2012048242A1

[22] Diego C de, Chen F, Xie Y, Pai RK, Slavin L, Parker J, et al Anisotropic conduction block and reentry in neonatal rat ventricular myocyte monolayers. Am J Physiol—Heart Circ Physiol. 2011;300: H271–H278. doi: 10.1152/ajpheart.00758.2009 21037233 10.1152/ajpheart.00758.2009 PMC3023258

[23] Schwan J, Kwaczala AT, Ryan TJ, Bartulos O, Ren Y, Sewanan LR, et al Anisotropic engineered heart tissue made from laser-cut decellularized myocardium. Sci Rep. 2016;6 doi: 10.1038/srep32068 27572147 10.1038/srep32068 PMC5004193

[24] Spach MS, Heidlage JF, Barr RC, Dolber PC. Cell size and communication: role in structural and electrical development and remodeling of the heart. Heart Rhythm. 2004;1: 500–515. doi: 10.1016/j.hrthm.2004.06.010 15851207 10.1016/j.hrthm.2004.06.010

[25] Kim JM, Bursac N, Henriquez CS. A computer model of engineered cardiac monolayers. Biophys J. 2010;98: 1762–1771. doi: 10.1016/j.bpj.2010.01.008 20441739 10.1016/j.bpj.2010.01.008 PMC2862160

[26] Jacquemet V, Henriquez CS. Loading effect of fibroblast-myocyte coupling on resting potential, impulse propagation, and repolarization: insights from a microstructure model. Am J Physiol—Heart Circ Physiol. 2008;294: H2040–H2052. doi: 10.1152/ajpheart.01298.2007 18310514 10.1152/ajpheart.01298.2007 PMC3292859

[27] Luo CH, Rudy Y. A model of the ventricular cardiac action potential. Depolarization, repolarization, and their interaction. Circ Res. 1991;68: 1501–1526. doi: 10.1161/01.RES.68.6.1501 1709839 10.1161/01.res.68.6.1501

[28] Kanakov OI, Osipov GV, Chan C-K, Kurths J. Cluster synchronization and spatio-temporal dynamics in networks of oscillatory and excitable Luo-Rudy cells. Chaos Woodbury N. 2007;17: 015111 doi: 10.1063/1.2437581 17411268 10.1063/1.2437581

[29] Kryukov AK, Petrov VS, Averyanova LS, Osipov GV, Chen W, Drugova O, et al Synchronization phenomena in mixed media of passive, excitable, and oscillatory cells. Chaos Interdiscip J Nonlinear Sci. 2008;18: 037129 doi: 10.1063/1.2956985 19045503 10.1063/1.2956985

[30] AUTO [Internet]. [cited 12 Jul 2017]. Available: http://indy.cs.concordia.ca/auto/

[31] Bayly PV, KenKnight BH, Rogers JM, Hillsley RE, Ideker RE, Smith WM. Estimation of conduction velocity vector fields from epicardial mapping data. IEEE Trans Biomed Eng. 1998;45: 563–571. doi: 10.1109/10.668746 9581054 10.1109/10.668746

[32] Korhonen T, Hänninen SL, Tavi P. Model of Excitation-Contraction Coupling of Rat Neonatal Ventricular Myocytes. Biophys J. 2009;96: 1189–1209. doi: 10.1016/j.bpj.2008.10.026 19186154 10.1016/j.bpj.2008.10.026 PMC2716686

[33] Paci M, Hyttinen J, Aalto-Setälä K, Severi S. Computational models of ventricular- and atrial-like human induced pluripotent stem cell derived cardiomyocytes. Ann Biomed Eng. 2013;41: 2334–2348. doi: 10.1007/s10439-013-0833-3 23722932 10.1007/s10439-013-0833-3

[34] Hui BBCB, Dokos S, Lovell NH. Parameter Identifiability of Cardiac Ionic Models Using a Novel CellML Least Squares Optimization Tool. 2007 29th Annual International Conference of the IEEE Engineering in Medicine and Biology Society. 2007. pp. 5307–5310. doi: 10.1109/IEMBS.2007.4353539 10.1109/IEMBS.2007.4353539 18003205

[35] Shajahan TK, Borek B, Shrier A, Glass L. Scaling properties of conduction velocity in heterogeneous excitable media. Phys Rev E Stat Nonlin Soft Matter Phys. 2011;84: 046208 doi: 10.1103/PhysRevE.84.046208 22181246 10.1103/PhysRevE.84.046208

[36] Unudurthi SD, Wolf RM, Hund TJ. Role of sinoatrial node architecture in maintaining a balanced source-sink relationship and synchronous cardiac pacemaking. Front Physiol. 2014;5 doi: 10.3389/fphys.2014.00446 25505419 10.3389/fphys.2014.00446 PMC4244803

[37] Bub G, Shrier A, Glass L. Global organization of dynamics in oscillatory heterogeneous excitable media. Phys Rev Lett. 2005;94: 028105 doi: 10.1103/PhysRevLett.94.028105 15698236 10.1103/PhysRevLett.94.028105

[38] Bub G, Shrier A, Glass L. Spiral wave generation in heterogeneous excitable media. Phys Rev Lett. 2002;88: 058101 doi: 10.1103/PhysRevLett.88.058101 11863783 10.1103/PhysRevLett.88.058101

[39] Steinberg BE, Glass L, Shrier A, Bub G. The role of heterogeneities and intercellular coupling in wave propagation in cardiac tissue. Philos Trans R Soc Lond Math Phys Eng Sci. 2006;364: 1299–1311. doi: 10.1098/rsta.2006.1771 16608709 10.1098/rsta.2006.1771

[40] Boyett MR, Inada S, Yoo S, Li J, Liu J, Tellez J, et al Connexins in the sinoatrial and atrioventricular nodes. Adv Cardiol. 2006;42: 175–197. doi: 10.1159/000092569 16646591 10.1159/000092569

[41] Kleber AG, Saffitz JE. Role of the intercalated disc in cardiac propagation and arrhythmogenesis. Front Physiol. 2014;5 doi: 10.3389/fphys.2014.00404 25368581 10.3389/fphys.2014.00404 PMC4201087

[42] Boudreau-Béland J, Duverger JE, Petitjean E, Maguy A, Ledoux J, Comtois P. Spatiotemporal Stability of Neonatal Rat Cardiomyocyte Monolayers Spontaneous Activity Is Dependent on the Culture Substrate. PLoS ONE. 2015;10 doi: 10.1371/journal.pone.0127977 26035822 10.1371/journal.pone.0127977 PMC4452796

[43] Ponard JGC, Kondratyev AA, Kucera JP. Mechanisms of Intrinsic Beating Variability in Cardiac Cell Cultures and Model Pacemaker Networks. Biophys J. 2007;92: 3734–3752. doi: 10.1529/biophysj.106.091892 17325022 10.1529/biophysj.106.091892 PMC1853135

[44] Pong T, Adams WJ, Bray M-A, Feinberg AW, Sheehy SP, Werdich AA, et al Hierarchical architecture influences calcium dynamics in engineered cardiac muscle. Exp Biol Med Maywood NJ. 2011;236: 366–373. doi: 10.1258/ebm.2010.010239 21330361 10.1258/ebm.2010.010239 PMC4501496

[45] Walsh KB, Parks GE. Changes in cardiac myocyte morphology alter the properties of voltage-gated ion channels. Cardiovasc Res. 2002;55: 64–75. 12062709 10.1016/s0008-6363(02)00403-0

[46] Li N, Hansen BJ, Csepe TA, Zhao J, Ignozzi AJ, Sul LV, et al Redundant and diverse intranodal pacemakers and conduction pathways protect the human sinoatrial node from failure. Sci Transl Med. 2017;9 doi: 10.1126/scitranslmed.aam5607 28747516 10.1126/scitranslmed.aam5607 PMC5775890

[47] Spach MS, Heidlage JF, Dolber PC, Barr RC. Electrophysiological Effects of Remodeling Cardiac Gap Junctions and Cell Size. Circ Res. 2000;86: 302–311. doi: 10.1161/01.RES.86.3.302 10679482 10.1161/01.res.86.3.302

[48] Xie Y, Sato D, Garfinkel A, Qu Z, Weiss JN. So Little Source, So Much Sink: Requirements for Afterdepolarizations to Propagate in Tissue. Biophys J. 2010;99: 1408–1415. doi: 10.1016/j.bpj.2010.06.042 20816052 10.1016/j.bpj.2010.06.042 PMC2931729

